# Conventional versus reverse sequence of neoadjuvant epirubicin/cyclophosphamide and docetaxel: sequencing results from ABCSG-34

**DOI:** 10.1038/s41416-021-01284-2

**Published:** 2021-03-24

**Authors:** Rupert Bartsch, Christian F. Singer, Georg Pfeiler, Michael Hubalek, Herbert Stoeger, Angelika Pichler, Edgar Petru, Vesna Bjelic-Radisic, Richard Greil, Margaretha Rudas, Tea Maria Muy-Kheng, Viktor Wette, Andreas L. Petzer, Paul Sevelda, Daniel Egle, Peter C. Dubsky, Martin Filipits, Florian Fitzal, Ruth Exner, Raimund Jakesz, Marija Balic, Christoph Tinchon, Zsuzsanna Bago-Horvath, Sophie Frantal, Michael Gnant, for the Austrian Breast and Colorectal Cancer Study Group

**Affiliations:** 1 Department of Medicine 1, Clinical Division of Oncology, Medical University of Vienna, Vienna, Austria; 2 Department of Gynecology, Medical University of Vienna, Vienna, Austria; 3 Breast Center Schwaz, BKH Schwaz, Schwaz, Austria; 4 Division of Oncology, Department of Internal Medicine and Comprehensive Cancer Center, Medical University of Graz, Graz, Austria; 5 Department of Hemato-Oncology, LKH Hochsteiermark-Leoben, Leoben, Austria; 6 Department of Gynecology and Obstetrics, Medical University of Graz, Graz, Austria; 7 Breast Unit, Helios University Hospital Wuppertal, Wuppertal Germany, University Witten/Herdecke, Wuppertal, Germany; 8 Department of Internal Medicine III with Hematology, Medical Oncology, Hemostaseology, Infectious Disease, Rheumatology, Oncologic Center, Laboratory for Immunological and Molecular Cancer Research, Paracelsus Medical University, Salzburg, Austria; 9 Department of Pathology, Medical University of Vienna, Vienna, Austria; 10 Breastcenter Carinthia, St. Veit, Austria; 11 Internal Medicine I, Hematology with Stem Cell Transplantation, Hemostaseology and Medical Oncology, Ordensklinikum Linz Barmherzige Schwestern, Elisabethinen, Linz, Austria; 12 Karl Landsteiner Institute for Gynecologic Oncology and Senology, Vienna, Austria; 13 Department of Obstetrics and Gynecology, Medical University of Innsbruck, Innsbruck, Austria; 14 Department of Surgery and Breast Health Center of the Comprehensive Cancer Center, Medical University of Vienna, Vienna, Austria; 15 Breastcenter St. Anna, Lucerne, Switzerland; 16 Institute of Cancer Research, Department of Medicine I, Comprehensive Cancer Center, Medical University of Vienna, Vienna, Austria; 17 Statistics Department, Austrian Breast & Colorectal Cancer Study Group (ABCSG), Vienna, Austria; 18 Comprehensive Cancer Center, Medical University Vienna, Vienna, Austria

**Keywords:** Breast cancer, Chemotherapy

## Abstract

**Background:**

Preoperative chemotherapy containing anthracyclines and taxanes is well established in early-stage breast cancer. Previous studies have suggested that the chemotherapy sequence may matter but definitive evidence is missing. ABCSG trial 34 evaluated the activity of the MUC1 vaccine tecemotide when added to neoadjuvant treatment; the study provided the opportunity for the second randomisation to compare two different anthracycline/taxane sequences.

**Methods:**

HER2-negative early-stage breast cancer patients were recruited to this randomised multicentre Phase 2 study. Patients in the chemotherapy cohort (*n* = 311) were additionally randomised to a conventional or reversed sequence of epirubicin/cyclophosphamide and docetaxel. Residual cancer burden (RCB) with/without tecemotide was defined as primary study endpoint; RCB in the two chemotherapy groups was a key secondary endpoint.

**Results:**

No significant differences in terms of RCB 0/I (40.1% vs. 37.2%; *P* = 0.61) or pathologic complete response (pCR) rates (24.3% vs. 25%, *P* = 0.89) were observed between conventional or reverse chemotherapy sequence. No new safety signals were reported, and upfront docetaxel did not result in decreased rates of treatment delay or discontinuation.

**Conclusion:**

Upfront docetaxel did not improve chemotherapy activity or tolerability; these results suggest that upfront neoadjuvant treatment with anthracyclines remains a valid option.

## Background

Preoperative chemotherapy was developed in patients with locally advanced, inoperable breast cancer.^1^ Today, the neoadjuvant administration of systemic treatment has turned into a standard option whenever chemotherapy is indicated in principle since preoperative therapy improves breast conservation rates and provides information on response and disease biology.^2^

National Surgical Breast and Bowel Project (NSABP) trial B-27 investigated the addition of four cycles of pre- or postoperative docetaxel to four cycles of AC (doxorubicin/cyclophosphamide).^3^ Neoadjuvant treatment with AC-docetaxel yielded a significant increase in pathologic complete remission (pCR) rate from 12.9% (AC) to 26.1% (AC-docetaxel) and pCR correlated with improved overall survival (OS). This led to further evaluations of the prognostic role of pCR as a surrogate endpoint and two metanalyses have since confirmed the relationship of pCR and long-term outcome on an individual patient level in high-risk breast cancer subtypes while achieving pCR is apparently of less relevance in luminal disease.^4,5^ pCR, however, dichotomises responses which may not fully reflect the true prognosis of patients as non-pCR includes outcomes ranging from minimal residual disease (MRD) to progression. Therefore, the residual cancer burden (RCB) score was developed, wherein RCB 0 reflects pCR or in situ disease only and RCB I reflects a minimal amount of residual invasive cancer with comparable long-term outcome.^6^

As shown in NSABP B-27, the conventional sequence of chemotherapeutic drugs is the upfront administration of anthracyclines and cyclophosphamide followed by a taxane.^3,7^ This was challenged by observations that upfront treatment with taxanes may improve outcome^8^ and tolerability,^9^ thereby renewing interest in the question of chemotherapy sequencing. Of note, preclinical studies support an upfront taxane approach as well.^10–13^

ABCSG-34 is a randomised Phase 2 study evaluating the addition of tecemotide (liposomal BLP25; L-BLP25; Stimuvax^®^), a MUC1 vaccine, to standard preoperative treatment consisting either of endocrine therapy or chemotherapy.^14^ In the chemotherapy cohort, participants were subjected to second randomisation comparing four cycles of docetaxel after (conventional sequence) or before four cycles of epirubicin/cyclophosphamide (EC) (reverse sequence). Here, we present results of the secondary chemotherapy-sequencing randomisation.

## Methods

### Study design

ABCSG-34 is an academic, prospective, randomised, open-label, multicentre, Phase 2 trial evaluating the efficacy and safety of tecemotide as a component of neoadjuvant therapy in HER2-negative early-stage breast cancer. While postmenopausal patients with luminal A-like tumours received preoperative endocrine treatment with six months of letrozole, premenopausal patients and patients with luminal B-like or triple-negative breast cancer (TNBC) were scheduled for preoperative chemotherapy. For this study, luminal A like was defined as follows: high or intermediate expression of the oestrogen receptor (ER), grade 1 or 2, and a proliferation rate <14%. A cut-off of <10% was chosen to define negative ER expression.

**Fig. 1:**
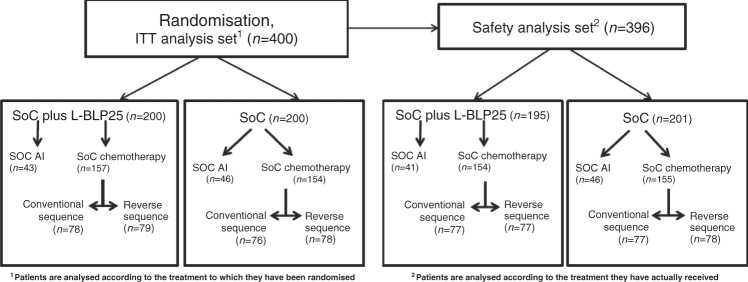
Consort diagram: ABCSG-34 trial overview. ITT intent-to-treat, SoC Standard-of-Care, AI aromatase inhibitor.

Patients accrued to the chemotherapy cohort were randomly assigned to a conventional chemotherapy sequence (four cycles of EC followed by four cycles of docetaxel) or to a reversed sequence thereof (upfront docetaxel followed by EC) (Consort diagram, Fig. 1).

The rate of patients with RCB 0/I score with or without tecemotide was defined as primary study endpoint; secondary endpoints included the rate of patients with RCB 0/I score in the two chemotherapy-sequencing arms, pCR rates, safety and quality-of-life (QoL) in patients with or without tecemotide.

### Patients

Women aged 18 and older with histologically proven, invasive HER2-negative breast cancer without evidence of distant metastases scheduled to receive preoperative therapy were eligible.

### Treatment

Patients accrued to the chemotherapy cohort received four consecutive cycles of EC (epirubicin 90 mg/m^2^ and cyclophosphamide 600 mg/m^2^ q3w), followed by four cycles of docetaxel (100 mg/m^2^, 3qw) (conventional sequence), or the reversed sequence thereof. In addition, patients randomised to tecemotide received a single i.v. infusion of cyclophosphamide (300 mg/m^2^) 3 days prior to the first vaccination.

### Response assessments

Pathological assessment of response to neoadjuvant treatment for the primary outcome analysis was performed by the RCB score;^6^ in short, the RCB score is a continuous variable based on the primary tumour bed dimensions, cellularity of the invasive tumour component, and axillary lymph node burden at the surgery. An RCB score of <1.36 describes minimal (RCB I) or absent (RCB 0) residual invasive disease. pCR was defined as ypT0/is ypN0 in the surgical specimen. RCB and pCR were both assessed locally after appropriate training of local pathologists; the quality of RCB 0/1 readouts was centrally reviewed by the ABCSG central trial pathologist.

Adverse events (AEs) and serious adverse events (SAEs) were classified according to World Health Organisation (WHO) criteria.

### Statistical analysis

Patients were randomised 1:1 to standard neoadjuvant therapy with or without tecemotide using minimisation. All analyses, except for the safety analysis, were based on the intention to treat (ITT) principle with patients analysed according to the treatment to which they had been randomised to (Fig. 1). Only randomised patients who were evaluable at the time of final surgery were included in the efficacy analysis. The safety analysis set included all randomised patients with at least one administration of study treatment. The overall sample-size calculation of ABCSG-34 was based upon the primary endpoint; no sample-size calculation for the second randomisation (conventional vs. reverse chemotherapy sequence) was performed.

Patient and treatment characteristics, as well as safety data (AEs and SAEs) were described descriptively per chemotherapy treatment arm. Comparisons of proportions of RCB and pCR between treatment arms were performed with the chi-square test. Multivariate logistic regression models were used to adjust response for demographic or prognostic factors. Odds ratios (OR) with 95% confidence interval (CI) are provided.

## Results

### Patient characteristics

A total number of 311 patients were accrued to the chemotherapy cohort. The median age was 49 years (25–78), 184 patients (59.2%) were premenopausal, 126 (40.5%) had node-positive tumours at diagnosis, 51.8% had oestrogen-receptor positive breast cancer and 37.3% TNBC, respectively. Table 1 lists the patient’s characteristics for the entire chemotherapy cohort as well as for the sequencing arms. Apart from nodal status, no major inhomogeneities were observed between the groups.

**Table 1:** Patient characteristics.

	Chemo conventional, *N* = 154	Chemo reverse, *N* = 157	Total, *N* = 311	*P* value
Age (years)				
*N*	154	157	311	
Mean	49.1	49.5	49.3	
SD	10.7	11.3	11.0	
Median	49.0	48.0	49.0	
Min	26.0	25.0	25.0	
Max	78.0	75.0	78.0	
Wilcoxon				0.8836
BMI				
*N*	153	156	309	
Mean	25.5	25.6	25.6	
SD	5.0	5.0	5.0	
Median	24.5	24.5	24.5	
Min	16.5	15.0	15.0	
Max	42.8	40.5	42.8	
Wilcoxon				0.8605
Menopausal status, *n* (%)^a^				
Perimenopausal	56 (36.4%)	56 (35.7%)	112 (36.0%)	
Postmenopausal	8 (5.2%)	3 (1.9%)	11 (3.5%)	
Premenopausal	89 (57.8%)	95 (60.5%)	184 (59.2%)	
Missing	1 (0.6%)	3 (1.9%)	4 (1.3%)	
Chi-square				0.2915
T-stage, *n* (%)				
T1	39 (25.3%)	47 (29.9%)	86 (27.7%)	
T2	99 (64.3%)	90 (57.3%)	189 (60.8%)	
T3	13 (8.4%)	18 (11.5%)	31 (10.0%)	
T4	3 (1.9%)	2 (1.3%)	5 (1.6%)	
Fisher				0.548
Triple negative, *n* (%)^b^				
No	91 (59.1%)	85 (54.1%)	176 (56.6%)	
Yes	54 (35.1%)	62 (39.5%)	116 (37.3%)	
Missing	9 (5.8%)	10 (6.4%)	19 (6.1%)	
Chi-square				0.3888
N-stage, *n* (%)				
Negative	94 (61.0%)	84 (53.5%)	178 (57.2%)	
Positive	57 (37.0%)	69 (43.9%)	126 (40.5%)	
Missing	3 (1.9%)	4 (2.5%)	7 (2.3%)	
Chi-square				0.1934
Grading, *n* (%)				
G1	1 (0.6%)	1 (0.6%)	2 (0.6%)	
G2/Gx	43 (27.9%)	49 (31.2%)	92 (29.6%)	
G3	107 (69.5%)	105 (66.9%)	212 (68.2%)	
Missing	3 (1.9%)	2 (1.3%)	5 (1.6%)	
Fisher				0.8083
HER2, *n* (%)				
Negative	132 (85.7%)	137 (87.3%)	269 (86.5%)	
Positive	1 (0.6%)	1 (0.6%)	2 (0.6%)	
Missing	21 (13.6%)	19 (12.1%)	40 (12.9%)	
Fisher				1
ER, *n* (%)				
Negative	68 (44.2%)	76 (48.4%)	144 (46.3%)	
Positive	83 (53.9%)	78 (49.7%)	161 (51.8%)	
Missing	3 (1.9%)	3 (1.9%)	6 (1.9%)	
Chi-square				0.4502
PgR, *n* (%)				
Negative	76 (49.4%)	81 (51.6%)	157 (50.5%)	
Positive	75 (48.7%)	74 (47.1%)	149 (47.9%)	
Missing	3 (1.9%)	2 (1.3%)	5 (1.6%)	
Chi-square				0.736
Ki67				
*N*	148	147	295	
Mean	50.4	49.6	50.0	
SD	24.3	23.9	24.1	
Median	50.0	50.0	50.0	
Min	2.0	3.0	2.0	
Max	90.0	95.0	95.0	
Wilcoxon				0.6744

*N* number of patients in the ITT analysis set, *n* number of patients, *SD*   standard deviation, *Min* minimum, *Max* maximum.

For patients with bilateral breast cancer, information from the higher disease stage is used for descriptive summaries.

^a^Women are considered postmenopausal if they have not had a menstrual period for >12 months due to natural causes or had a bilateral oophorectomy, and/or have serum levels of oestradiol, LH and FSH within the postmenopausal range.

^b^TNBC includes patients who have negative ER, PR and Her2 status—patients with missing information in any of these variables are not considered as TNBC.

In the overall chemotherapy cohort, 95.2% of patients had completed treatment as planned per protocol. Respective numbers are 95.5% in the conventional sequence arm and 94.9% in patients receiving upfront docetaxel. The mean number of chemotherapy cycles was 7.4 and again comparable (7.5 conventional sequence arm; 7.3 reverse sequence).

### Residual cancer burden (RCB) and pathological complete remission (pCR) in patients treated with conventional or reversed chemotherapy sequence

**Fig. 2:**
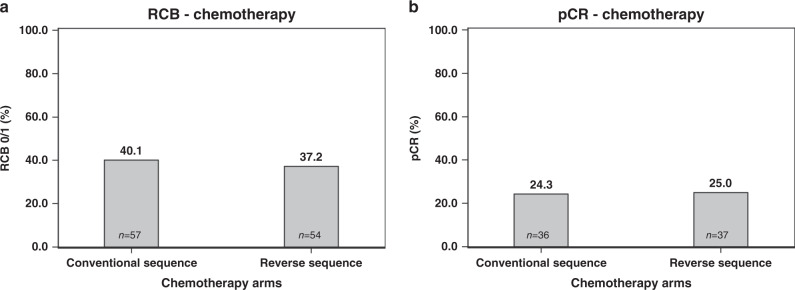
RCB 0/1 rate and pCR rate in patients with conventional and reverse chemotherapy sequence. RCB residual cancer burden, pCR pathologic complete remission. Conventional vs. reverse in chemotherapy patients (**a**) RCB 0/I rate, (**b**) pCR rate.

In patients with conventional chemotherapy sequence, an RCB 0/1 rate of 40.1% (*n* = 57) was recorded as compared with 37.2% (*n* = 54) in the upfront docetaxel group (*P* = 0.61, chi-square test, Fig. 2a). Respective numbers for pCR rates were 24.3% (*n* = 36, upfront anthracyclines) and 25.0% (*n* = 37, upfront docetaxel) (*P* = 0.89) (Fig. 2b).

Separate post hoc analyses of surgical outcome conducted in patients with TNBC and luminal breast cancer revealed minor outcome disparities between the two chemotherapy arms. In TNBC, a numerical benefit was observed in terms of RCB 0/1 and pCR rates favouring the conventional sequence group (RCB 0/1 rate conventional sequence 55.8% vs. reverse sequence 48.3%; pCR rate conventional sequence 45.3% vs. reverse sequence 31.2%), while numerically superior results with reverse chemotherapy sequence were observed in the in luminal breast cohort (RCB 0/1 rate conventional sequence 19.7% vs. reverse sequence 25.0%; pCR rate conventional sequence 1.6% vs. reverse sequence 14.5%), respectively.

### Residual cancer burden (RCB) and pathological complete remission (pCR) in patients treated with conventional or reversed chemotherapy sequence with/without tecemotide

**Fig. 3:**
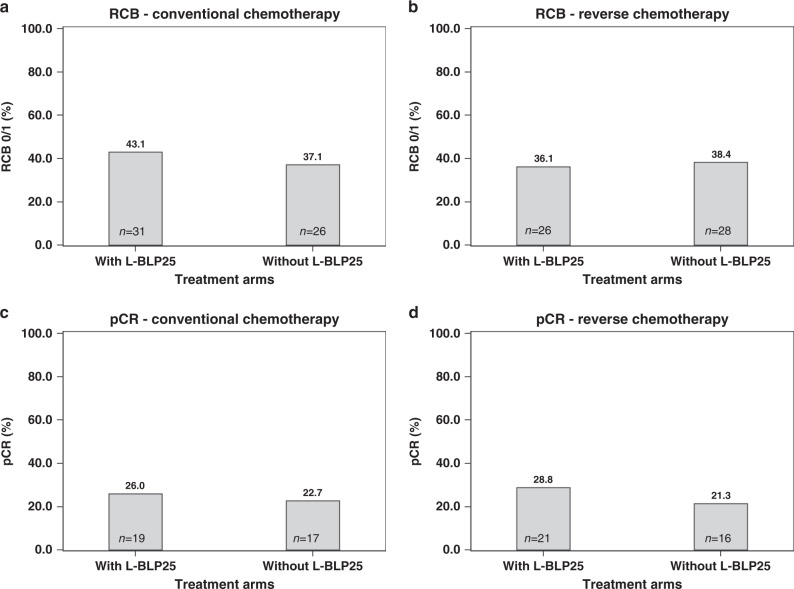
RCB 0/1 rate and pCR rate in patients with conventional and reverse chemotherapy sequence with or without L-BLP25. RCB residual cancer burden, pCR pathologic complete remission. With vs. without L-BLP25 (**a**) conventional chemotherapy patients and RCB 0/I rate, (**b**) reverse chemotherapy patients and RCB 0/I rate, (**c**) conventional chemotherapy patients and pCR rate, (**d**) reverse chemotherapy patients and pCR rate.

Regarding outcome in the overall chemotherapy cohort, no difference in terms of RCB 0/1 score was observed between patients with or without tecemotide (39.6% vs. 37.8%; *P* = 0.75, chi-square test). In the conventional sequence arm, an RCB score of 0/1 was recorded in 43.1% (*n* = 31) of patients with tecemotide as compared with 37.1% (*n* = 26) of patients without; in the upfront docetaxel group, corresponding numbers were 36.1% (*n* = 26) with and 38.4% (*n* = 28) without tecemotide, respectively (Fig. 3a, b).

A pCR was observed in 27.4% and 22.0% of patients with or without tecemotide in the chemotherapy cohort (*P* = 0.2815, chi-square test). When analysing the two sequencing arms separately, pCR rates were 26.0% (*n* = 19) and 22.7% (*n* = 17) in the conventional sequence arm in patients with or without tecemotide; corresponding numbers in the reverse sequence arm were 28.8% (*n* = 21) and 21.3% (*n* = 16), respectively (Fig. 3c, d).

Based on covariate analyses, within the subset of patients receiving chemotherapy, parameters significantly associated with RCB 0/I were Ki67 (OR 0.98; 95% CI 0.97–0.99) and progesterone receptor status (reference are negative patients; OR 0.24; 95% CI 0.09–0.64). No influence of tecemotide or chemotherapy sequence on outcome was detected in these models.

### Safety

The safety population in the chemotherapy cohort consisted of 309 patients who had received at least one dose of study medication.

All patients had at least one AE in both treatment arms; regarding grade 3/4 AEs, corresponding numbers are 58.4% in the conventional and 52.3% in the reverse sequence arm, respectively. A numerically higher rate of neutropenia was observed with the conventional chemotherapy sequence (51/154 vs. 44/155), febrile neutropenia, however, was more common in the reverse sequence arm (13/155 vs. 3/154). Regarding non-haematological side effects, a slightly higher rate of eye disorders was observed with upfront docetaxel (56/155 *vs*. 43/154), while nausea was more common with upfront EC (108/154 vs. 91/155), as was cough (25/154 vs. 13/155). Skin disorders, again, were more commonly reported with upfront docetaxel (121/155 vs. 105/154). Grade 3/4 side effects occurring in ≥5 patients are summarised in Table 2; a complete list of AEs of patients in either sequencing arms is provided in Supplementary Table 1.

**Table 2:** Grade 3/4 AEs occurring in ≥5 patients.

by SOC and PT	Chemo conventional with L-BLP25, *N* = 154	Chemo reverse with L-BLP25, *N* = 155	Total, *N* = 309
Number of patients with at least one grade 3/4 AE, *n* (%)			
	90 (58.4%)	81 (52.3%)	171 (55.3%)
Blood and lymphatic system disorders, *n* (%)			
Febrile neutropenia	3 (1.9%)	13 (8.4%)	16 (5.2%)
Leukopenia	41 (26.6%)	29 (18.7%)	70 (22.7%)
Neutropenia	50 (32.5%)	37 (23.9%)	87 (28.2%)
Gastrointestinal disorders, *n* (%)			
Diarrhoea	2 (1.3%)	5 (3.2%)	7 (2.3%)
General disorders and administration site conditions, *n* (%)			
Asthenia	3 (1.9%)	2 (1.3%)	5 (1.6%)
Fatigue	3 (1.9%)	3 (1.9%)	6 (1.9%)
Investigations, *n* (%)			
Neutrophil count decreased	2 (1.3%)	3 (1.9%)	5 (1.6%)
Musculoskeletal and connective tissue disorders, *n* (%)			
Bone pain	3 (1.9%)	2 (1.3%)	5 (1.6%)
Myalgia	3 (1.9%)	2 (1.3%)	5 (1.6%)
Respiratory, thoracic and mediastinal disorders, *n* (%)			
Pulmonary embolism	1 (0.6%)	4 (2.6%)	5 (1.6%)

*N* number of patients in the safety analysis set, *n* number of patients, *SOC* system organ class, *PT* preferred term.

A numerically higher SAE rate was observed in the reverse sequence arm (54/155 (34.8%) vs. 41/154 (26.6%)). Overall, three cases of pulmonary embolism were observed (all in the reverse sequence group). SAEs occurring in ≥5 patients for the two sequencing arms are summarised in Table 3. Two deaths occurred on the trial (pulmonary embolism, *n* = 1; breast cancer progression, *n* = 1), both in the reverse sequence arm while none was observed in the upfront EC group.

**Table 3:** SAEs occurring in ≥5 patients.

by SOC and PT	Chemo conventional, *N* = 154	Chemo reverse, *N* = 155	Total, *N* = 309
Number of patients with at least one SAE^a^, *n* (%)			
	41 (26.6%)	54 (34.8%)	95 (30.7%)
Blood and lymphatic system disorders, *n* (%)			
Febrile neutropenia	2 (1.3%)	12 (7.7%)	14 (4.5%)
Leukopenia	4 (2.6%)	4 (2.6%)	8 (2.6%)
Neutropenia	4 (2.6%)	4 (2.6%)	8 (2.6%)
Gastrointestinal disorders, *n* (%)			
Diarrhoea	5 (3.2%)	4 (2.6%)	9 (2.9%)
General disorders and administration site conditions, *n* (%)			
Pyrexia	4 (2.6%)	1 (0.6%)	5 (1.6%)
Musculoskeletal and connective tissue disorders, *n* (%)			
Bone pain	2 (1.3%)	3 (1.9%)	5 (1.6%)

*N* number of patients in the safety analysis set, *n* number of patients, *SOC*   system organ class, *PT* preferred term.

^a^SAE: any adverse event resulting in death, is immediately life-threatening, requires inpatient hospitalisation or prolongation of hospitalisation, results in persistent or significant disability/incapacity, is a congenital anomaly/birth defect in a child whose parent was exposed to a medicinal product prior to conception or during pregnancy or is considered otherwise medically significant such as important medical events that may not immediately be life-threatening or result in death or hospitalisation, but jeopardise the subject or require intervention to prevent one of the outcomes listed in the definition above.

### Dose modifications, treatment delays and treatment discontinuations

With regards to chemotherapy discontinuation rates, 9/154 (5.8%) and 10/157 (6.4%) patients stopped docetaxel due to AEs in the conventional and reverse sequence arms, respectively; numerically more patients in the upfront docetaxel arm stopped taxane-based chemotherapy due to disease progression (4/157 vs. 1/154). In contrast, EC was discontinued due to disease progression in only two patients (both in the reverse sequence arm). EC was discontinued due to AEs in three patients, two in the conventional sequence arm and one with reverse chemotherapy sequence, respectively.

In the upfront EC group, more patients received epirubicin and cyclophosphamide without dose delays (60.4% vs. 38.9% epirubicin; 59.7% vs. 39.5% cyclophosphamide) while the rate of docetaxel dose delays was numerically lower in the reverse sequence arm (patients without dose delays conventional sequence 44.8% vs. reverse sequence 52.9%); regarding dose modifications, slightly more patients received EC without dose modifications in the conventional sequence arm (89.0% vs. 82.2%), while the rate of patients without dose modifications of docetaxel was numerically higher in the upfront taxane group (conventional sequence 66.9% vs. reverse sequence 79.6%).

## Discussion

The prospective, randomised Phase 2 trial ABCSG-34 evaluated the addition of the MUC1 vaccine tecemotide to neoadjuvant treatment.^14^ In total, 311 patients were accrued to the chemotherapy cohort and randomised to either a conventional chemotherapy sequence of EC followed by docetaxel or a reverse sequence with upfront docetaxel, making this the largest randomised trial evaluating the role of EC/docetaxel sequencing in the neoadjuvant setting in a HER2-negative population to date (while Neo-tAnGo was the largest sequencing study in general). In our study, no significant efficacy differences were observed, and tolerability was comparable as well. These results need to be discussed in the light of experimental data as well as results of other studies evaluating chemotherapy sequencing in the neoadjuvant and adjuvant setting.

A cell culture model suggested that MCF-7 breast cancer cells resistant to doxorubicin were cross-resistant to paclitaxel and docetaxel due to an upregulation of P-glycoprotein; in a model of paclitaxel resistance, however, only limited cross-resistance to doxorubicin was observed.^10^ Anthracycline-induced senescence, cellular response to nonlethal stress resulting in persistent cytostasis, may constitute another reason for the assumed reduced activity of taxanes when administered after anthracyclines.^11,12^ In addition, in a study evaluating levels of circulating tumour cells (CTC) in patients receiving neoadjuvant chemotherapy, a decrease of CTC levels was observed during the initial anthracycline treatment phase. This was followed by a resurgence of CTC levels during the paclitaxel phase suggestive of resistance despite a further reduction in primary tumour size.^13^

In metastatic breast cancer, a prospective randomised Phase 3 trial compared the combination of doxorubicin plus paclitaxel to either doxorubicin or paclitaxel alone with a pre-specified crossover at the time of progression.^15^ No difference in terms of response rates, time to treatment failure and overall survival was observed between the two sequential arms. Furthermore, response rates after crossover were similar as well (20% in patients crossing-over from doxorubicin to paclitaxel and 22% with paclitaxel to doxorubicin, respectively). These results, however, may be impacted on by the well-established heterogeneity of the metastatic disease.^16^

In the neoadjuvant setting, the prospective randomised Phase 3 trial Neo-tAnGo randomised 831 patients to four cycles of EC followed by four cycles of dose-dense paclitaxel (with or without gemcitabine) or the reverse sequence thereof. Around one-quarter of patients were HER2-positive. In this study, upfront paclitaxel treatment resulted in significantly higher pCR rates (15% vs. 20%; *P* = 0.03), while no significant difference in terms of disease-free survival and OS was observed.^17^ Regarding tolerability, more dose reductions, and dose delays in the fourth cycle of paclitaxel were observed when anthracyclines were administered first (22% vs. 10% and 15% vs. 11%, respectively). These results are supported by data from a retrospective analysis of 1414 patients were also significantly higher pCR rates were reported when paclitaxel was administered before FEC/FAC (20.9% vs. 12.4%; *P* = 0.04).^8^ In contrast, no pCR increase was reported with upfront paclitaxel in a Phase 3 trial conducted in HER2-positive early-stage breast cancer; participants, however, received additional immunotherapy with trastuzumab, potentially reducing the relative importance of chemotherapy.^18^ Three small, randomised Phase 2 studies investigating doxorubicin followed by paclitaxel^19,20^ or weekly docetaxel^21^ or the reverse sequence thereof could not establish a significant improvement of response rates with the reversed sequence as well. Moreover, a French trial randomising 123 patients to four cycles of docetaxel followed by four cycles of anthracycline-containing chemotherapy or vice versa yielded results similar to our study.^22^ No difference in terms of response or breast conservation rate was observed but a higher neurotoxicity rate was reported in patients with upfront docetaxel (which was not seen in ABCSG-34).

Except for one small study,^23^ the majority of sequencing trials conducted in the adjuvant setting consistently suggested improved tolerability when taxanes were administered first, resulting in a higher relative chemotherapy dose-intensity and less treatment delays.^9,24,25^ Due to the size and design of these trials, however, no data regarding the influence of chemotherapy sequencing on long-term outcomes are available.

Results of ABCSG-34 are therefore in line with data from several smaller neoadjuvant studies suggesting that despite the preclinical rationale, upfront taxane therapy may not increase pCR rates in early-stage breast cancer. Of note, these studies often used suboptimal chemotherapy regimens (e.g., cyclophosphamide-free, weekly docetaxel). In contrast, the largest sequencing study hitherto, neo-tAnGo, reported contradicting results: here, a significantly higher pCR rate was observed when paclitaxel was administered first. Some relevant differences, however, need be kept in mind: Other than in ABCSG-34, a quarter of the study population participating in neo-tAnGo was HER2-positive; also, paclitaxel was chosen as taxane backbone and gemcitabine was added to half the subjects which may have altered tolerability. A Cochrane review investigating anthracycline/taxane sequencing yielded results similar to our trial: data from 1415 participants in five neoadjuvant trials, among them neo-tAnGo, were analysed and upfront administration of taxanes resulted in little to no difference in terms of OS, DFS, and pCR.^26^ neo-tAnGo and several other studies reported improved tolerability as indicted by fewer dose reductions and fewer dose delays with upfront taxane administration. This was not mirrored in ABCSG-34 where tolerability was overall comparable between the arms, and even a higher febrile neutropenia rate with upfront docetaxel was observed which may be explained by the numerically lower rate of docetaxel dose reductions in this group. Again, these differences might be due to the fact that docetaxel 100 mg/m² once every 3 weeks was chosen as chemotherapy backbone in ABCSG-34, and the sequence of anthracycline and taxanes may be more relevant when using paclitaxel and/or dose-dense regimens.

In ACSCG-34, none of the patients in the conventional sequence arm discontinued upfront EC for disease progression as opposed to 2.6% discontinuing upfront docetaxel in the reverse sequence arm. While these numbers are small, they are in line with results from the prospective randomised Phase 3 GeparSepto trial, where a discontinuation rate of 2% and 5% was reported with upfront neoadjuvant nab-paclitaxel and conventional paclitaxel, respectively.^27^ Further investigation of the primary progression rate in studies using an upfront taxane design may therefore be warranted. More recently, however, the upfront combination of paclitaxel and carboplatin became widely used in TNBC, thereby reducing the relevance of this issue.^28^

A major limitation of the trial is the fact that the secondary sequencing randomisation was not stratified for baseline characteristics such as subtype and nodal status, and no separate sample-size calculation was performed. This has led to an imbalance in the baseline characteristics between the two sequencing arms with a higher rate of N + patients in the reversed sequence group (44% and 37%, respectively); in addition, small patient numbers in the subgroups of TNBC and luminal breast cancer patients may have resulted in the numerically different effects of chemotherapy sequences in terms of RCB 0/1 and pCR rates. A further limitation is the lack of survival follow-up. Therefore, the results must be interpreted with due caution despite being in line with a recent Cochrane review.

Despite these limitations, the prospective randomised Phase 2 trial ABCSG-34 is to date the largest randomised trial evaluating the effect of EC/docetaxel sequencing in the neoadjuvant setting in a HER2-negative population. This study did not observe the hypothesised benefit of upfront docetaxel administration in terms of chemotherapy activity. In addition, tolerability was not improved as well. While no final conclusions can be drawn, upfront administration of anthracyclines followed by docetaxel, therefore, remains a potential treatment standard and results of this trial suggest that clinicians may choose upfront administration of EC or docetaxel as preferred.

## Supplementary information

Supplementary Table 1

## Data Availability

As the regulatory sponsor of this trial, ABCSG has data sovereignty and individual participant data (including de-identified participant data, participant data with identifiers, data dictionary or other specified datasets) will not be shared.
