# Supplementary material for: Conventional versus reverse sequence of neoadjuvant epirubicin/cyclophosphamide and docetaxel: sequencing results from ABCSG-34
Source: Br J Cancer. 2021 Mar 24;124(11):1795–802. doi: 10.1038/s41416-021-01284-2 (PMC8144560; doi:10.1038/s41416-021-01284-2)
Supplement: Supplementary file 1 — Supplementary Table 1 [file 41416_2021_1284_MOESM1_ESM.docx]

**Supplementary Table 1**

All AEs

| *by SOC and PT* | *Chemo conventional with L-BLP25 N=77* | *Chemo reverse with L-BLP25 N=77* | *Chemo conventional without L-BLP25 N=77* | *Chemo reverse without L-BLP25 N=78* | *Total N=309* |  |  | |
| --- | --- | --- | --- | --- | --- | --- | --- | --- |
| **Number of Patients with At Least One AE** | | | | | | | |  |
|  | 77 (100.0%) | 77 (100.0%) | 77 (100.0%) | 78 (100.0%) | 309 (100.0%) |  |  | |
|  | | | | | | | |  |
| **Blood and lymphatic system disorders** | | | | | | | |  |
|  | 37 (48.1%) | 39 (50.6%) | 38 (49.4%) | 36 (46.2%) | 150 (48.5%) |  |  | |
| Anaemia | 10 (13.0%) | 9 (11.7%) | 17 (22.1%) | 13 (16.7%) | 49 (15.9%) |  |  | |
| Febrile neutropenia | 2 (2.6%) | 7 (9.1%) | 1 (1.3%) | 6 (7.7%) | 16 (5.2%) |  |  | |
| Leukocytosis | 0 (0.0%) | 0 (0.0%) | 1 (1.3%) | 1 (1.3%) | 2 (0.6%) |  |  | |
| Leukopenia | 25 (32.5%) | 19 (24.7%) | 25 (32.5%) | 15 (19.2%) | 84 (27.2%) |  |  | |
| Lymphadenopathy | 1 (1.3%) | 1 (1.3%) | 0 (0.0%) | 0 (0.0%) | 2 (0.6%) |  |  | |
| Neutropenia | 27 (35.1%) | 26 (33.8%) | 24 (31.2%) | 18 (23.1%) | 95 (30.7%) |  |  | |
| Pancytopenia | 1 (1.3%) | 0 (0.0%) | 0 (0.0%) | 0 (0.0%) | 1 (0.3%) |  |  | |
| Thrombocytopenia | 2 (2.6%) | 0 (0.0%) | 0 (0.0%) | 1 (1.3%) | 3 (1.0%) |  |  | |
| Thrombocytosis | 1 (1.3%) | 0 (0.0%) | 0 (0.0%) | 0 (0.0%) | 1 (0.3%) |  |  | |
|  | | | | | | | |  |
| **Cardiac disorders** | | | | | | | |  |
|  | 14 (18.2%) | 12 (15.6%) | 8 (10.4%) | 12 (15.4%) | 46 (14.9%) |  |  | |
| Angina pectoris | 0 (0.0%) | 0 (0.0%) | 0 (0.0%) | 1 (1.3%) | 1 (0.3%) |  |  | |
| Arrhythmia | 0 (0.0%) | 1 (1.3%) | 0 (0.0%) | 0 (0.0%) | 1 (0.3%) |  |  | |
| Atrial fibrillation | 1 (1.3%) | 0 (0.0%) | 0 (0.0%) | 0 (0.0%) | 1 (0.3%) |  |  | |
| Cardiac disorder | 1 (1.3%) | 0 (0.0%) | 0 (0.0%) | 0 (0.0%) | 1 (0.3%) |  |  | |
| Cardiovascular disorder | 4 (5.2%) | 2 (2.6%) | 3 (3.9%) | 1 (1.3%) | 10 (3.2%) |  |  | |
| Cardiovascular insufficiency | 1 (1.3%) | 1 (1.3%) | 1 (1.3%) | 2 (2.6%) | 5 (1.6%) |  |  | |
| Palpitations | 1 (1.3%) | 2 (2.6%) | 0 (0.0%) | 2 (2.6%) | 5 (1.6%) |  |  | |
| Sinus tachycardia | 1 (1.3%) | 0 (0.0%) | 0 (0.0%) | 1 (1.3%) | 2 (0.6%) |  |  | |
| Supraventricular tachycardia | 0 (0.0%) | 0 (0.0%) | 0 (0.0%) | 1 (1.3%) | 1 (0.3%) |  |  | |
| Tachycardia | 6 (7.8%) | 6 (7.8%) | 5 (6.5%) | 4 (5.1%) | 21 (6.8%) |  |  | |
|  | | | | | | | |  |
| **Congenital, familial and genetic disorders** | | | | | | | |  |
|  | 0 (0.0%) | 1 (1.3%) | 2 (2.6%) | 1 (1.3%) | 4 (1.3%) |  |  | |
| Epidermolysis | 0 (0.0%) | 0 (0.0%) | 1 (1.3%) | 0 (0.0%) | 1 (0.3%) |  |  | |
| Factor V deficiency | 0 (0.0%) | 0 (0.0%) | 0 (0.0%) | 1 (1.3%) | 1 (0.3%) |  |  | |
| Sebaceous naevus | 0 (0.0%) | 0 (0.0%) | 1 (1.3%) | 0 (0.0%) | 1 (0.3%) |  |  | |
| Umbilical haematoma | 0 (0.0%) | 1 (1.3%) | 0 (0.0%) | 0 (0.0%) | 1 (0.3%) |  |  | |
|  | | | | | | | |  |
| **Ear and labyrinth disorders** | | | | | | | |  |
|  | 18 (23.4%) | 23 (29.9%) | 13 (16.9%) | 13 (16.7%) | 67 (21.7%) |  |  | |
| Cerumen impaction | 0 (0.0%) | 0 (0.0%) | 1 (1.3%) | 0 (0.0%) | 1 (0.3%) |  |  | |
| Deafness | 0 (0.0%) | 1 (1.3%) | 0 (0.0%) | 0 (0.0%) | 1 (0.3%) |  |  | |
| Deafness neurosensory | 0 (0.0%) | 0 (0.0%) | 0 (0.0%) | 1 (1.3%) | 1 (0.3%) |  |  | |
| Ear pain | 1 (1.3%) | 1 (1.3%) | 1 (1.3%) | 3 (3.8%) | 6 (1.9%) |  |  | |
| Hearing impaired | 2 (2.6%) | 1 (1.3%) | 1 (1.3%) | 0 (0.0%) | 4 (1.3%) |  |  | |
| Hypoacusis | 0 (0.0%) | 0 (0.0%) | 1 (1.3%) | 0 (0.0%) | 1 (0.3%) |  |  | |
| Neurosensory hypoacusis | 0 (0.0%) | 1 (1.3%) | 0 (0.0%) | 0 (0.0%) | 1 (0.3%) |  |  | |
| Ototoxicity | 0 (0.0%) | 1 (1.3%) | 2 (2.6%) | 2 (2.6%) | 5 (1.6%) |  |  | |
| Tinnitus | 2 (2.6%) | 4 (5.2%) | 0 (0.0%) | 0 (0.0%) | 6 (1.9%) |  |  | |
| Vertigo | 14 (18.2%) | 18 (23.4%) | 10 (13.0%) | 7 (9.0%) | 49 (15.9%) |  |  | |
|  | | | | | | | |  |
| **Endocrine disorders** | | | | | | | |  |
|  | 0 (0.0%) | 1 (1.3%) | 2 (2.6%) | 0 (0.0%) | 3 (1.0%) |  |  | |
| Hyperthyroidism | 0 (0.0%) | 1 (1.3%) | 1 (1.3%) | 0 (0.0%) | 2 (0.6%) |  |  | |
| Hypothyroidism | 0 (0.0%) | 0 (0.0%) | 1 (1.3%) | 0 (0.0%) | 1 (0.3%) |  |  | |
|  | | | | | | | |  |
| **Eye disorders** | | | | | | | |  |
|  | 16 (20.8%) | 29 (37.7%) | 27 (35.1%) | 27 (34.6%) | 99 (32.0%) |  |  | |
| Amblyopia | 1 (1.3%) | 1 (1.3%) | 0 (0.0%) | 2 (2.6%) | 4 (1.3%) |  |  | |
| Blepharitis | 0 (0.0%) | 1 (1.3%) | 0 (0.0%) | 0 (0.0%) | 1 (0.3%) |  |  | |
| Blepharospasm | 0 (0.0%) | 0 (0.0%) | 0 (0.0%) | 1 (1.3%) | 1 (0.3%) |  |  | |
| Chalazion | 0 (0.0%) | 1 (1.3%) | 0 (0.0%) | 0 (0.0%) | 1 (0.3%) |  |  | |
| Conjunctivitis | 2 (2.6%) | 5 (6.5%) | 1 (1.3%) | 2 (2.6%) | 10 (3.2%) |  |  | |
| Cyanopsia | 1 (1.3%) | 0 (0.0%) | 0 (0.0%) | 0 (0.0%) | 1 (0.3%) |  |  | |
| Dacryostenosis acquired | 1 (1.3%) | 0 (0.0%) | 0 (0.0%) | 0 (0.0%) | 1 (0.3%) |  |  | |
| Diabetic retinopathy | 0 (0.0%) | 1 (1.3%) | 0 (0.0%) | 0 (0.0%) | 1 (0.3%) |  |  | |
| Dry eye | 5 (6.5%) | 5 (6.5%) | 3 (3.9%) | 4 (5.1%) | 17 (5.5%) |  |  | |
| Eye discharge | 0 (0.0%) | 0 (0.0%) | 1 (1.3%) | 0 (0.0%) | 1 (0.3%) |  |  | |
| Eye irritation | 0 (0.0%) | 1 (1.3%) | 0 (0.0%) | 1 (1.3%) | 2 (0.6%) |  |  | |
| Eye pain | 0 (0.0%) | 0 (0.0%) | 0 (0.0%) | 1 (1.3%) | 1 (0.3%) |  |  | |
| Eye pruritus | 0 (0.0%) | 0 (0.0%) | 1 (1.3%) | 0 (0.0%) | 1 (0.3%) |  |  | |
| Eye swelling | 1 (1.3%) | 0 (0.0%) | 0 (0.0%) | 0 (0.0%) | 1 (0.3%) |  |  | |
| Eyelid oedema | 1 (1.3%) | 1 (1.3%) | 1 (1.3%) | 2 (2.6%) | 5 (1.6%) |  |  | |
| Keratitis | 0 (0.0%) | 1 (1.3%) | 0 (0.0%) | 0 (0.0%) | 1 (0.3%) |  |  | |
| Lacrimation increased | 8 (10.4%) | 18 (23.4%) | 17 (22.1%) | 15 (19.2%) | 58 (18.8%) |  |  | |
| Mydriasis | 0 (0.0%) | 1 (1.3%) | 0 (0.0%) | 0 (0.0%) | 1 (0.3%) |  |  | |
| Ocular hyperaemia | 0 (0.0%) | 1 (1.3%) | 0 (0.0%) | 0 (0.0%) | 1 (0.3%) |  |  | |
| Panophthalmitis | 0 (0.0%) | 0 (0.0%) | 1 (1.3%) | 2 (2.6%) | 3 (1.0%) |  |  | |
| Periorbital oedema | 0 (0.0%) | 1 (1.3%) | 0 (0.0%) | 0 (0.0%) | 1 (0.3%) |  |  | |
| Photophobia | 0 (0.0%) | 0 (0.0%) | 1 (1.3%) | 0 (0.0%) | 1 (0.3%) |  |  | |
| Photopsia | 0 (0.0%) | 0 (0.0%) | 1 (1.3%) | 0 (0.0%) | 1 (0.3%) |  |  | |
| Vision blurred | 0 (0.0%) | 0 (0.0%) | 1 (1.3%) | 2 (2.6%) | 3 (1.0%) |  |  | |
| Visual acuity reduced | 1 (1.3%) | 0 (0.0%) | 5 (6.5%) | 1 (1.3%) | 7 (2.3%) |  |  | |
| Visual impairment | 0 (0.0%) | 1 (1.3%) | 0 (0.0%) | 1 (1.3%) | 2 (0.6%) |  |  | |
|  | | | | | | | |  |
| **Gastrointestinal disorders** | | | | | | | |  |
|  | 67 (87.0%) | 69 (89.6%) | 70 (90.9%) | 61 (78.2%) | 267 (86.4%) |  |  | |
| Abdominal discomfort | 3 (3.9%) | 5 (6.5%) | 0 (0.0%) | 4 (5.1%) | 12 (3.9%) |  |  | |
| Abdominal distension | 0 (0.0%) | 3 (3.9%) | 0 (0.0%) | 0 (0.0%) | 3 (1.0%) |  |  | |
| Abdominal pain | 6 (7.8%) | 5 (6.5%) | 2 (2.6%) | 4 (5.1%) | 17 (5.5%) |  |  | |
| Abdominal pain lower | 1 (1.3%) | 0 (0.0%) | 0 (0.0%) | 0 (0.0%) | 1 (0.3%) |  |  | |
| Abdominal pain upper | 5 (6.5%) | 8 (10.4%) | 7 (9.1%) | 6 (7.7%) | 26 (8.4%) |  |  | |
| Abnormal faeces | 0 (0.0%) | 1 (1.3%) | 0 (0.0%) | 1 (1.3%) | 2 (0.6%) |  |  | |
| Anal fissure | 0 (0.0%) | 0 (0.0%) | 0 (0.0%) | 1 (1.3%) | 1 (0.3%) |  |  | |
| Anal haemorrhage | 1 (1.3%) | 0 (0.0%) | 0 (0.0%) | 0 (0.0%) | 1 (0.3%) |  |  | |
| Anorectal discomfort | 1 (1.3%) | 0 (0.0%) | 0 (0.0%) | 0 (0.0%) | 1 (0.3%) |  |  | |
| Aphthous stomatitis | 1 (1.3%) | 2 (2.6%) | 0 (0.0%) | 1 (1.3%) | 4 (1.3%) |  |  | |
| Aptyalism | 0 (0.0%) | 0 (0.0%) | 1 (1.3%) | 0 (0.0%) | 1 (0.3%) |  |  | |
| Bowel movement irregularity | 0 (0.0%) | 1 (1.3%) | 1 (1.3%) | 0 (0.0%) | 2 (0.6%) |  |  | |
| Cheilitis | 0 (0.0%) | 0 (0.0%) | 1 (1.3%) | 0 (0.0%) | 1 (0.3%) |  |  | |
| Colitis | 0 (0.0%) | 0 (0.0%) | 1 (1.3%) | 0 (0.0%) | 1 (0.3%) |  |  | |
| Colonic obstruction | 0 (0.0%) | 0 (0.0%) | 0 (0.0%) | 1 (1.3%) | 1 (0.3%) |  |  | |
| Constipation | 27 (35.1%) | 27 (35.1%) | 27 (35.1%) | 30 (38.5%) | 111 (35.9%) |  |  | |
| Diarrhoea | 18 (23.4%) | 30 (39.0%) | 23 (29.9%) | 29 (37.2%) | 100 (32.4%) |  |  | |
| Diverticulum intestinal haemorrhagic | 0 (0.0%) | 0 (0.0%) | 0 (0.0%) | 1 (1.3%) | 1 (0.3%) |  |  | |
| Dry mouth | 6 (7.8%) | 2 (2.6%) | 6 (7.8%) | 1 (1.3%) | 15 (4.9%) |  |  | |
| Duodenal ulcer haemorrhage | 0 (0.0%) | 1 (1.3%) | 0 (0.0%) | 0 (0.0%) | 1 (0.3%) |  |  | |
| Duodenogastric reflux | 0 (0.0%) | 0 (0.0%) | 0 (0.0%) | 1 (1.3%) | 1 (0.3%) |  |  | |
| Dyspepsia | 6 (7.8%) | 7 (9.1%) | 6 (7.8%) | 5 (6.4%) | 24 (7.8%) |  |  | |
| Dysphagia | 2 (2.6%) | 4 (5.2%) | 3 (3.9%) | 0 (0.0%) | 9 (2.9%) |  |  | |
| Enteritis | 0 (0.0%) | 1 (1.3%) | 0 (0.0%) | 0 (0.0%) | 1 (0.3%) |  |  | |
| Faecaloma | 0 (0.0%) | 1 (1.3%) | 0 (0.0%) | 0 (0.0%) | 1 (0.3%) |  |  | |
| Faeces discoloured | 1 (1.3%) | 0 (0.0%) | 0 (0.0%) | 0 (0.0%) | 1 (0.3%) |  |  | |
| Flatulence | 2 (2.6%) | 0 (0.0%) | 4 (5.2%) | 1 (1.3%) | 7 (2.3%) |  |  | |
| Gastric disorder | 1 (1.3%) | 0 (0.0%) | 0 (0.0%) | 0 (0.0%) | 1 (0.3%) |  |  | |
| Gastric hypertonia | 0 (0.0%) | 0 (0.0%) | 1 (1.3%) | 0 (0.0%) | 1 (0.3%) |  |  | |
| Gastritis | 1 (1.3%) | 0 (0.0%) | 0 (0.0%) | 0 (0.0%) | 1 (0.3%) |  |  | |
| Gastritis erosive | 0 (0.0%) | 0 (0.0%) | 1 (1.3%) | 0 (0.0%) | 1 (0.3%) |  |  | |
| Gastrointestinal disorder | 0 (0.0%) | 0 (0.0%) | 0 (0.0%) | 1 (1.3%) | 1 (0.3%) |  |  | |
| Gastrointestinal pain | 0 (0.0%) | 0 (0.0%) | 0 (0.0%) | 1 (1.3%) | 1 (0.3%) |  |  | |
| Gastrointestinal sounds abnormal | 0 (0.0%) | 0 (0.0%) | 1 (1.3%) | 0 (0.0%) | 1 (0.3%) |  |  | |
| Gastrooesophageal reflux disease | 6 (7.8%) | 3 (3.9%) | 5 (6.5%) | 3 (3.8%) | 17 (5.5%) |  |  | |
| Gingival swelling | 1 (1.3%) | 0 (0.0%) | 0 (0.0%) | 0 (0.0%) | 1 (0.3%) |  |  | |
| Gingivitis | 3 (3.9%) | 1 (1.3%) | 1 (1.3%) | 2 (2.6%) | 7 (2.3%) |  |  | |
| Glossitis | 0 (0.0%) | 0 (0.0%) | 0 (0.0%) | 1 (1.3%) | 1 (0.3%) |  |  | |
| Glossodynia | 2 (2.6%) | 0 (0.0%) | 0 (0.0%) | 0 (0.0%) | 2 (0.6%) |  |  | |
| Haematochezia | 2 (2.6%) | 0 (0.0%) | 0 (0.0%) | 0 (0.0%) | 2 (0.6%) |  |  | |
| Haemorrhoids | 4 (5.2%) | 2 (2.6%) | 1 (1.3%) | 1 (1.3%) | 8 (2.6%) |  |  | |
| Hypoaesthesia oral | 0 (0.0%) | 1 (1.3%) | 0 (0.0%) | 0 (0.0%) | 1 (0.3%) |  |  | |
| Lip dry | 0 (0.0%) | 0 (0.0%) | 0 (0.0%) | 1 (1.3%) | 1 (0.3%) |  |  | |
| Lip swelling | 1 (1.3%) | 0 (0.0%) | 0 (0.0%) | 0 (0.0%) | 1 (0.3%) |  |  | |
| Loose tooth | 0 (0.0%) | 0 (0.0%) | 0 (0.0%) | 1 (1.3%) | 1 (0.3%) |  |  | |
| Nausea | 57 (74.0%) | 50 (64.9%) | 51 (66.2%) | 41 (52.6%) | 199 (64.4%) |  |  | |
| Oesophageal ulcer | 0 (0.0%) | 1 (1.3%) | 0 (0.0%) | 0 (0.0%) | 1 (0.3%) |  |  | |
| Oesophagitis | 1 (1.3%) | 0 (0.0%) | 0 (0.0%) | 0 (0.0%) | 1 (0.3%) |  |  | |
| Oral disorder | 0 (0.0%) | 1 (1.3%) | 0 (0.0%) | 0 (0.0%) | 1 (0.3%) |  |  | |
| Oral dysaesthesia | 1 (1.3%) | 0 (0.0%) | 0 (0.0%) | 0 (0.0%) | 1 (0.3%) |  |  | |
| Oral pain | 1 (1.3%) | 0 (0.0%) | 1 (1.3%) | 0 (0.0%) | 2 (0.6%) |  |  | |
| Palatitis | 0 (0.0%) | 0 (0.0%) | 0 (0.0%) | 1 (1.3%) | 1 (0.3%) |  |  | |
| Paraesthesia oral | 1 (1.3%) | 0 (0.0%) | 0 (0.0%) | 0 (0.0%) | 1 (0.3%) |  |  | |
| Periodontal disease | 0 (0.0%) | 0 (0.0%) | 0 (0.0%) | 1 (1.3%) | 1 (0.3%) |  |  | |
| Periodontitis | 0 (0.0%) | 0 (0.0%) | 1 (1.3%) | 0 (0.0%) | 1 (0.3%) |  |  | |
| Rectal haemorrhage | 0 (0.0%) | 0 (0.0%) | 1 (1.3%) | 0 (0.0%) | 1 (0.3%) |  |  | |
| Salivary hypersecretion | 0 (0.0%) | 1 (1.3%) | 0 (0.0%) | 0 (0.0%) | 1 (0.3%) |  |  | |
| Stomatitis | 24 (31.2%) | 17 (22.1%) | 15 (19.5%) | 18 (23.1%) | 74 (23.9%) |  |  | |
| Swollen tongue | 2 (2.6%) | 0 (0.0%) | 0 (0.0%) | 0 (0.0%) | 2 (0.6%) |  |  | |
| Tongue blistering | 1 (1.3%) | 0 (0.0%) | 0 (0.0%) | 0 (0.0%) | 1 (0.3%) |  |  | |
| Tongue coated | 0 (0.0%) | 2 (2.6%) | 1 (1.3%) | 0 (0.0%) | 3 (1.0%) |  |  | |
| Tongue discolouration | 0 (0.0%) | 1 (1.3%) | 1 (1.3%) | 0 (0.0%) | 2 (0.6%) |  |  | |
| Tooth discolouration | 0 (0.0%) | 1 (1.3%) | 0 (0.0%) | 0 (0.0%) | 1 (0.3%) |  |  | |
| Tooth disorder | 0 (0.0%) | 0 (0.0%) | 1 (1.3%) | 0 (0.0%) | 1 (0.3%) |  |  | |
| Tooth loss | 0 (0.0%) | 0 (0.0%) | 0 (0.0%) | 1 (1.3%) | 1 (0.3%) |  |  | |
| Toothache | 3 (3.9%) | 2 (2.6%) | 1 (1.3%) | 3 (3.8%) | 9 (2.9%) |  |  | |
| Vomiting | 17 (22.1%) | 6 (7.8%) | 15 (19.5%) | 10 (12.8%) | 48 (15.5%) |  |  | |
|  | | | | | | | |  |
| **General disorders and administration site conditions** | | | | | | | |  |
|  | 68 (88.3%) | 69 (89.6%) | 60 (77.9%) | 62 (79.5%) | 259 (83.8%) |  |  | |
| Application site erythema | 0 (0.0%) | 0 (0.0%) | 0 (0.0%) | 1 (1.3%) | 1 (0.3%) |  |  | |
| Asthenia | 3 (3.9%) | 7 (9.1%) | 7 (9.1%) | 3 (3.8%) | 20 (6.5%) |  |  | |
| Axillary pain | 4 (5.2%) | 3 (3.9%) | 2 (2.6%) | 1 (1.3%) | 10 (3.2%) |  |  | |
| Catheter site erythema | 0 (0.0%) | 0 (0.0%) | 1 (1.3%) | 0 (0.0%) | 1 (0.3%) |  |  | |
| Catheter site haemorrhage | 0 (0.0%) | 1 (1.3%) | 0 (0.0%) | 0 (0.0%) | 1 (0.3%) |  |  | |
| Catheter site inflammation | 0 (0.0%) | 0 (0.0%) | 0 (0.0%) | 1 (1.3%) | 1 (0.3%) |  |  | |
| Catheter site pain | 2 (2.6%) | 1 (1.3%) | 2 (2.6%) | 0 (0.0%) | 5 (1.6%) |  |  | |
| Catheter site related reaction | 0 (0.0%) | 0 (0.0%) | 1 (1.3%) | 0 (0.0%) | 1 (0.3%) |  |  | |
| Catheter site swelling | 0 (0.0%) | 0 (0.0%) | 0 (0.0%) | 1 (1.3%) | 1 (0.3%) |  |  | |
| Chest discomfort | 2 (2.6%) | 3 (3.9%) | 1 (1.3%) | 2 (2.6%) | 8 (2.6%) |  |  | |
| Chest pain | 4 (5.2%) | 4 (5.2%) | 2 (2.6%) | 2 (2.6%) | 12 (3.9%) |  |  | |
| Chills | 3 (3.9%) | 3 (3.9%) | 1 (1.3%) | 2 (2.6%) | 9 (2.9%) |  |  | |
| Exercise tolerance decreased | 1 (1.3%) | 0 (0.0%) | 0 (0.0%) | 0 (0.0%) | 1 (0.3%) |  |  | |
| Extravasation | 2 (2.6%) | 0 (0.0%) | 0 (0.0%) | 1 (1.3%) | 3 (1.0%) |  |  | |
| Face oedema | 2 (2.6%) | 1 (1.3%) | 0 (0.0%) | 1 (1.3%) | 4 (1.3%) |  |  | |
| Fatigue | 54 (70.1%) | 45 (58.4%) | 49 (63.6%) | 47 (60.3%) | 195 (63.1%) |  |  | |
| Feeling abnormal | 0 (0.0%) | 0 (0.0%) | 0 (0.0%) | 1 (1.3%) | 1 (0.3%) |  |  | |
| Feeling cold | 3 (3.9%) | 1 (1.3%) | 1 (1.3%) | 2 (2.6%) | 7 (2.3%) |  |  | |
| Feeling hot | 0 (0.0%) | 1 (1.3%) | 0 (0.0%) | 0 (0.0%) | 1 (0.3%) |  |  | |
| Gait disturbance | 0 (0.0%) | 1 (1.3%) | 0 (0.0%) | 0 (0.0%) | 1 (0.3%) |  |  | |
| General physical health deterioration | 1 (1.3%) | 2 (2.6%) | 1 (1.3%) | 0 (0.0%) | 4 (1.3%) |  |  | |
| Generalised oedema | 0 (0.0%) | 1 (1.3%) | 0 (0.0%) | 0 (0.0%) | 1 (0.3%) |  |  | |
| Hyperthermia | 1 (1.3%) | 0 (0.0%) | 0 (0.0%) | 0 (0.0%) | 1 (0.3%) |  |  | |
| Impaired healing | 0 (0.0%) | 0 (0.0%) | 1 (1.3%) | 0 (0.0%) | 1 (0.3%) |  |  | |
| Implant site extravasation | 0 (0.0%) | 0 (0.0%) | 1 (1.3%) | 0 (0.0%) | 1 (0.3%) |  |  | |
| Implant site pain | 0 (0.0%) | 0 (0.0%) | 1 (1.3%) | 0 (0.0%) | 1 (0.3%) |  |  | |
| Induration | 0 (0.0%) | 2 (2.6%) | 0 (0.0%) | 0 (0.0%) | 2 (0.6%) |  |  | |
| Inflammation | 0 (0.0%) | 1 (1.3%) | 0 (0.0%) | 0 (0.0%) | 1 (0.3%) |  |  | |
| Influenza like illness | 1 (1.3%) | 3 (3.9%) | 1 (1.3%) | 3 (3.8%) | 8 (2.6%) |  |  | |
| Infusion site extravasation | 0 (0.0%) | 0 (0.0%) | 1 (1.3%) | 0 (0.0%) | 1 (0.3%) |  |  | |
| Infusion site pain | 0 (0.0%) | 0 (0.0%) | 0 (0.0%) | 1 (1.3%) | 1 (0.3%) |  |  | |
| Infusion site rash | 1 (1.3%) | 0 (0.0%) | 0 (0.0%) | 0 (0.0%) | 1 (0.3%) |  |  | |
| Injection site erythema | 4 (5.2%) | 9 (11.7%) | 0 (0.0%) | 0 (0.0%) | 13 (4.2%) |  |  | |
| Injection site haematoma | 0 (0.0%) | 1 (1.3%) | 0 (0.0%) | 0 (0.0%) | 1 (0.3%) |  |  | |
| Injection site induration | 1 (1.3%) | 3 (3.9%) | 0 (0.0%) | 0 (0.0%) | 4 (1.3%) |  |  | |
| Injection site pain | 3 (3.9%) | 1 (1.3%) | 0 (0.0%) | 1 (1.3%) | 5 (1.6%) |  |  | |
| Injection site reaction | 2 (2.6%) | 1 (1.3%) | 0 (0.0%) | 0 (0.0%) | 3 (1.0%) |  |  | |
| Injury associated with device | 1 (1.3%) | 0 (0.0%) | 0 (0.0%) | 0 (0.0%) | 1 (0.3%) |  |  | |
| Localised oedema | 0 (0.0%) | 0 (0.0%) | 0 (0.0%) | 1 (1.3%) | 1 (0.3%) |  |  | |
| Malaise | 1 (1.3%) | 1 (1.3%) | 0 (0.0%) | 3 (3.8%) | 5 (1.6%) |  |  | |
| Medical device complication | 0 (0.0%) | 0 (0.0%) | 1 (1.3%) | 1 (1.3%) | 2 (0.6%) |  |  | |
| Meteoropathy | 0 (0.0%) | 0 (0.0%) | 0 (0.0%) | 1 (1.3%) | 1 (0.3%) |  |  | |
| Mucosal dryness | 9 (11.7%) | 8 (10.4%) | 6 (7.8%) | 2 (2.6%) | 25 (8.1%) |  |  | |
| Mucosal inflammation | 4 (5.2%) | 6 (7.8%) | 6 (7.8%) | 7 (9.0%) | 23 (7.4%) |  |  | |
| Necrosis | 1 (1.3%) | 0 (0.0%) | 0 (0.0%) | 0 (0.0%) | 1 (0.3%) |  |  | |
| Nodule | 0 (0.0%) | 0 (0.0%) | 0 (0.0%) | 1 (1.3%) | 1 (0.3%) |  |  | |
| Oedema | 0 (0.0%) | 6 (7.8%) | 2 (2.6%) | 1 (1.3%) | 9 (2.9%) |  |  | |
| Oedema peripheral | 19 (24.7%) | 20 (26.0%) | 14 (18.2%) | 24 (30.8%) | 77 (24.9%) |  |  | |
| Pain | 10 (13.0%) | 7 (9.1%) | 12 (15.6%) | 3 (3.8%) | 32 (10.4%) |  |  | |
| Puncture site pain | 0 (0.0%) | 0 (0.0%) | 0 (0.0%) | 1 (1.3%) | 1 (0.3%) |  |  | |
| Pyrexia | 19 (24.7%) | 8 (10.4%) | 8 (10.4%) | 11 (14.1%) | 46 (14.9%) |  |  | |
| Sensation of foreign body | 0 (0.0%) | 1 (1.3%) | 0 (0.0%) | 0 (0.0%) | 1 (0.3%) |  |  | |
| Sense of oppression | 0 (0.0%) | 0 (0.0%) | 0 (0.0%) | 1 (1.3%) | 1 (0.3%) |  |  | |
| Temperature intolerance | 0 (0.0%) | 1 (1.3%) | 0 (0.0%) | 1 (1.3%) | 2 (0.6%) |  |  | |
| Thirst | 1 (1.3%) | 0 (0.0%) | 0 (0.0%) | 0 (0.0%) | 1 (0.3%) |  |  | |
| Thrombosis in device | 0 (0.0%) | 1 (1.3%) | 0 (0.0%) | 0 (0.0%) | 1 (0.3%) |  |  | |
| Ulcer | 0 (0.0%) | 0 (0.0%) | 1 (1.3%) | 0 (0.0%) | 1 (0.3%) |  |  | |
| Vaccination site erythema | 1 (1.3%) | 1 (1.3%) | 0 (0.0%) | 0 (0.0%) | 2 (0.6%) |  |  | |
| Vaccination site reaction | 1 (1.3%) | 0 (0.0%) | 0 (0.0%) | 0 (0.0%) | 1 (0.3%) |  |  | |
|  | | | | | | | |  |
| **Hepatobiliary disorders** | | | | | | | |  |
|  | 1 (1.3%) | 0 (0.0%) | 0 (0.0%) | 1 (1.3%) | 2 (0.6%) |  |  | |
| Hepatic pain | 1 (1.3%) | 0 (0.0%) | 0 (0.0%) | 0 (0.0%) | 1 (0.3%) |  |  | |
| Hepatic steatosis | 0 (0.0%) | 0 (0.0%) | 0 (0.0%) | 1 (1.3%) | 1 (0.3%) |  |  | |
|  | | | | | | | |  |
| **Immune system disorders** | | | | | | | |  |
|  | 3 (3.9%) | 4 (5.2%) | 3 (3.9%) | 2 (2.6%) | 12 (3.9%) |  |  | |
| Drug hypersensitivity | 2 (2.6%) | 1 (1.3%) | 1 (1.3%) | 1 (1.3%) | 5 (1.6%) |  |  | |
| Hypersensitivity | 1 (1.3%) | 3 (3.9%) | 2 (2.6%) | 1 (1.3%) | 7 (2.3%) |  |  | |
|  | | | | | | | |  |
| **Infections and infestations** | | | | | | | |  |
|  | 49 (63.6%) | 45 (58.4%) | 47 (61.0%) | 41 (52.6%) | 182 (58.9%) |  |  | |
| Abscess jaw | 0 (0.0%) | 0 (0.0%) | 1 (1.3%) | 0 (0.0%) | 1 (0.3%) |  |  | |
| Abscess limb | 1 (1.3%) | 0 (0.0%) | 0 (0.0%) | 0 (0.0%) | 1 (0.3%) |  |  | |
| Acute tonsillitis | 0 (0.0%) | 0 (0.0%) | 2 (2.6%) | 0 (0.0%) | 2 (0.6%) |  |  | |
| Appendicitis | 0 (0.0%) | 0 (0.0%) | 1 (1.3%) | 0 (0.0%) | 1 (0.3%) |  |  | |
| Bartholin's abscess | 0 (0.0%) | 1 (1.3%) | 0 (0.0%) | 0 (0.0%) | 1 (0.3%) |  |  | |
| Breast infection | 0 (0.0%) | 0 (0.0%) | 1 (1.3%) | 0 (0.0%) | 1 (0.3%) |  |  | |
| Bronchitis | 2 (2.6%) | 1 (1.3%) | 4 (5.2%) | 2 (2.6%) | 9 (2.9%) |  |  | |
| Bronchopneumonia | 0 (0.0%) | 1 (1.3%) | 0 (0.0%) | 1 (1.3%) | 2 (0.6%) |  |  | |
| Candidiasis | 2 (2.6%) | 0 (0.0%) | 0 (0.0%) | 1 (1.3%) | 3 (1.0%) |  |  | |
| Cystitis | 1 (1.3%) | 1 (1.3%) | 0 (0.0%) | 0 (0.0%) | 2 (0.6%) |  |  | |
| Dermatophytosis | 0 (0.0%) | 0 (0.0%) | 0 (0.0%) | 1 (1.3%) | 1 (0.3%) |  |  | |
| Device related infection | 1 (1.3%) | 0 (0.0%) | 0 (0.0%) | 2 (2.6%) | 3 (1.0%) |  |  | |
| Device related sepsis | 0 (0.0%) | 0 (0.0%) | 1 (1.3%) | 0 (0.0%) | 1 (0.3%) |  |  | |
| Erysipelas | 0 (0.0%) | 0 (0.0%) | 0 (0.0%) | 1 (1.3%) | 1 (0.3%) |  |  | |
| Febrile infection | 0 (0.0%) | 0 (0.0%) | 1 (1.3%) | 1 (1.3%) | 2 (0.6%) |  |  | |
| Folliculitis | 2 (2.6%) | 2 (2.6%) | 0 (0.0%) | 0 (0.0%) | 4 (1.3%) |  |  | |
| Fungal infection | 0 (0.0%) | 0 (0.0%) | 1 (1.3%) | 0 (0.0%) | 1 (0.3%) |  |  | |
| Furuncle | 1 (1.3%) | 0 (0.0%) | 0 (0.0%) | 0 (0.0%) | 1 (0.3%) |  |  | |
| Gastroenteritis | 0 (0.0%) | 0 (0.0%) | 1 (1.3%) | 0 (0.0%) | 1 (0.3%) |  |  | |
| Gastroenteritis viral | 0 (0.0%) | 0 (0.0%) | 0 (0.0%) | 1 (1.3%) | 1 (0.3%) |  |  | |
| Gastrointestinal infection | 1 (1.3%) | 1 (1.3%) | 1 (1.3%) | 0 (0.0%) | 3 (1.0%) |  |  | |
| Genital herpes | 1 (1.3%) | 0 (0.0%) | 0 (0.0%) | 1 (1.3%) | 2 (0.6%) |  |  | |
| Genital infection | 0 (0.0%) | 0 (0.0%) | 1 (1.3%) | 0 (0.0%) | 1 (0.3%) |  |  | |
| Genitourinary tract infection | 0 (0.0%) | 1 (1.3%) | 0 (0.0%) | 1 (1.3%) | 2 (0.6%) |  |  | |
| Helminthic infection | 0 (0.0%) | 0 (0.0%) | 1 (1.3%) | 0 (0.0%) | 1 (0.3%) |  |  | |
| Herpes simplex | 0 (0.0%) | 0 (0.0%) | 1 (1.3%) | 0 (0.0%) | 1 (0.3%) |  |  | |
| Herpes simplex ophthalmic | 0 (0.0%) | 0 (0.0%) | 1 (1.3%) | 0 (0.0%) | 1 (0.3%) |  |  | |
| Herpes virus infection | 1 (1.3%) | 0 (0.0%) | 1 (1.3%) | 0 (0.0%) | 2 (0.6%) |  |  | |
| Herpes zoster | 2 (2.6%) | 0 (0.0%) | 0 (0.0%) | 2 (2.6%) | 4 (1.3%) |  |  | |
| Hordeolum | 0 (0.0%) | 1 (1.3%) | 0 (0.0%) | 0 (0.0%) | 1 (0.3%) |  |  | |
| Implant site abscess | 0 (0.0%) | 0 (0.0%) | 1 (1.3%) | 0 (0.0%) | 1 (0.3%) |  |  | |
| Infection | 6 (7.8%) | 3 (3.9%) | 1 (1.3%) | 1 (1.3%) | 11 (3.6%) |  |  | |
| Influenza | 0 (0.0%) | 1 (1.3%) | 0 (0.0%) | 0 (0.0%) | 1 (0.3%) |  |  | |
| Laryngitis | 0 (0.0%) | 0 (0.0%) | 0 (0.0%) | 2 (2.6%) | 2 (0.6%) |  |  | |
| Localised infection | 0 (0.0%) | 0 (0.0%) | 0 (0.0%) | 1 (1.3%) | 1 (0.3%) |  |  | |
| Lyme disease | 1 (1.3%) | 0 (0.0%) | 0 (0.0%) | 0 (0.0%) | 1 (0.3%) |  |  | |
| Nail infection | 0 (0.0%) | 0 (0.0%) | 1 (1.3%) | 1 (1.3%) | 2 (0.6%) |  |  | |
| Nasopharyngitis | 16 (20.8%) | 8 (10.4%) | 13 (16.9%) | 10 (12.8%) | 47 (15.2%) |  |  | |
| Onychomycosis | 1 (1.3%) | 1 (1.3%) | 1 (1.3%) | 0 (0.0%) | 3 (1.0%) |  |  | |
| Oral candidiasis | 7 (9.1%) | 10 (13.0%) | 8 (10.4%) | 10 (12.8%) | 35 (11.3%) |  |  | |
| Oral fungal infection | 0 (0.0%) | 0 (0.0%) | 1 (1.3%) | 0 (0.0%) | 1 (0.3%) |  |  | |
| Oral herpes | 8 (10.4%) | 2 (2.6%) | 4 (5.2%) | 2 (2.6%) | 16 (5.2%) |  |  | |
| Parotitis | 1 (1.3%) | 0 (0.0%) | 0 (0.0%) | 0 (0.0%) | 1 (0.3%) |  |  | |
| Pharyngitis | 2 (2.6%) | 2 (2.6%) | 3 (3.9%) | 6 (7.7%) | 13 (4.2%) |  |  | |
| Pneumonia | 1 (1.3%) | 2 (2.6%) | 0 (0.0%) | 1 (1.3%) | 4 (1.3%) |  |  | |
| Postoperative wound infection | 0 (0.0%) | 0 (0.0%) | 1 (1.3%) | 1 (1.3%) | 2 (0.6%) |  |  | |
| Pulpitis dental | 0 (0.0%) | 1 (1.3%) | 0 (0.0%) | 0 (0.0%) | 1 (0.3%) |  |  | |
| Rash pustular | 0 (0.0%) | 1 (1.3%) | 0 (0.0%) | 0 (0.0%) | 1 (0.3%) |  |  | |
| Respiratory tract infection | 5 (6.5%) | 4 (5.2%) | 6 (7.8%) | 4 (5.1%) | 19 (6.1%) |  |  | |
| Respiratory tract infection viral | 0 (0.0%) | 1 (1.3%) | 0 (0.0%) | 0 (0.0%) | 1 (0.3%) |  |  | |
| Rhinitis | 5 (6.5%) | 8 (10.4%) | 8 (10.4%) | 7 (9.0%) | 28 (9.1%) |  |  | |
| Sepsis | 0 (0.0%) | 1 (1.3%) | 0 (0.0%) | 0 (0.0%) | 1 (0.3%) |  |  | |
| Sinusitis | 2 (2.6%) | 1 (1.3%) | 0 (0.0%) | 1 (1.3%) | 4 (1.3%) |  |  | |
| Tonsillitis | 0 (0.0%) | 1 (1.3%) | 0 (0.0%) | 1 (1.3%) | 2 (0.6%) |  |  | |
| Upper respiratory tract infection | 0 (0.0%) | 0 (0.0%) | 0 (0.0%) | 1 (1.3%) | 1 (0.3%) |  |  | |
| Urethritis | 1 (1.3%) | 0 (0.0%) | 0 (0.0%) | 0 (0.0%) | 1 (0.3%) |  |  | |
| Urinary tract infection | 8 (10.4%) | 5 (6.5%) | 7 (9.1%) | 5 (6.4%) | 25 (8.1%) |  |  | |
| Vaginal infection | 0 (0.0%) | 0 (0.0%) | 1 (1.3%) | 0 (0.0%) | 1 (0.3%) |  |  | |
| Viral infection | 1 (1.3%) | 1 (1.3%) | 1 (1.3%) | 0 (0.0%) | 3 (1.0%) |  |  | |
| Viral upper respiratory tract infection | 1 (1.3%) | 0 (0.0%) | 1 (1.3%) | 0 (0.0%) | 2 (0.6%) |  |  | |
| Vulvitis | 0 (0.0%) | 0 (0.0%) | 0 (0.0%) | 1 (1.3%) | 1 (0.3%) |  |  | |
| Vulvovaginal candidiasis | 1 (1.3%) | 1 (1.3%) | 1 (1.3%) | 1 (1.3%) | 4 (1.3%) |  |  | |
| Vulvovaginal mycotic infection | 2 (2.6%) | 1 (1.3%) | 1 (1.3%) | 3 (3.8%) | 7 (2.3%) |  |  | |
| Wound infection | 0 (0.0%) | 2 (2.6%) | 0 (0.0%) | 0 (0.0%) | 2 (0.6%) |  |  | |
|  | | | | | | | |  |
| **Injury, poisoning and procedural complications** | | | | | | | |  |
|  | 9 (11.7%) | 10 (13.0%) | 14 (18.2%) | 12 (15.4%) | 45 (14.6%) |  |  | |
| Arthropod bite | 0 (0.0%) | 1 (1.3%) | 0 (0.0%) | 2 (2.6%) | 3 (1.0%) |  |  | |
| Contusion | 0 (0.0%) | 1 (1.3%) | 0 (0.0%) | 0 (0.0%) | 1 (0.3%) |  |  | |
| Excoriation | 0 (0.0%) | 0 (0.0%) | 1 (1.3%) | 0 (0.0%) | 1 (0.3%) |  |  | |
| Eyelid injury | 0 (0.0%) | 0 (0.0%) | 0 (0.0%) | 1 (1.3%) | 1 (0.3%) |  |  | |
| Fractured sacrum | 0 (0.0%) | 0 (0.0%) | 1 (1.3%) | 0 (0.0%) | 1 (0.3%) |  |  | |
| Gingival injury | 0 (0.0%) | 0 (0.0%) | 1 (1.3%) | 0 (0.0%) | 1 (0.3%) |  |  | |
| Muscle strain | 0 (0.0%) | 0 (0.0%) | 0 (0.0%) | 1 (1.3%) | 1 (0.3%) |  |  | |
| Open wound | 1 (1.3%) | 0 (0.0%) | 0 (0.0%) | 0 (0.0%) | 1 (0.3%) |  |  | |
| Post procedural haemorrhage | 0 (0.0%) | 1 (1.3%) | 0 (0.0%) | 0 (0.0%) | 1 (0.3%) |  |  | |
| Procedural nausea | 0 (0.0%) | 0 (0.0%) | 0 (0.0%) | 1 (1.3%) | 1 (0.3%) |  |  | |
| Procedural pain | 2 (2.6%) | 3 (3.9%) | 4 (5.2%) | 4 (5.1%) | 13 (4.2%) |  |  | |
| Pubis fracture | 0 (0.0%) | 0 (0.0%) | 1 (1.3%) | 0 (0.0%) | 1 (0.3%) |  |  | |
| Seroma | 3 (3.9%) | 3 (3.9%) | 7 (9.1%) | 5 (6.4%) | 18 (5.8%) |  |  | |
| Wound | 0 (0.0%) | 1 (1.3%) | 0 (0.0%) | 0 (0.0%) | 1 (0.3%) |  |  | |
| Wound complication | 2 (2.6%) | 0 (0.0%) | 2 (2.6%) | 0 (0.0%) | 4 (1.3%) |  |  | |
| Wound dehiscence | 1 (1.3%) | 0 (0.0%) | 0 (0.0%) | 1 (1.3%) | 2 (0.6%) |  |  | |
| Wound secretion | 0 (0.0%) | 1 (1.3%) | 1 (1.3%) | 1 (1.3%) | 3 (1.0%) |  |  | |
|  | | | | | | | |  |
| **Investigations** | | | | | | | |  |
|  | 16 (20.8%) | 15 (19.5%) | 17 (22.1%) | 14 (17.9%) | 62 (20.1%) |  |  | |
| Alanine aminotransferase increased | 1 (1.3%) | 1 (1.3%) | 3 (3.9%) | 1 (1.3%) | 6 (1.9%) |  |  | |
| Aspartate aminotransferase increased | 1 (1.3%) | 1 (1.3%) | 3 (3.9%) | 0 (0.0%) | 5 (1.6%) |  |  | |
| Blood alkaline phosphatase increased | 2 (2.6%) | 0 (0.0%) | 0 (0.0%) | 0 (0.0%) | 2 (0.6%) |  |  | |
| Blood creatinine increased | 0 (0.0%) | 0 (0.0%) | 0 (0.0%) | 1 (1.3%) | 1 (0.3%) |  |  | |
| Blood glucose increased | 1 (1.3%) | 0 (0.0%) | 0 (0.0%) | 0 (0.0%) | 1 (0.3%) |  |  | |
| Blood lactate dehydrogenase increased | 0 (0.0%) | 0 (0.0%) | 1 (1.3%) | 0 (0.0%) | 1 (0.3%) |  |  | |
| Blood pressure decreased | 0 (0.0%) | 0 (0.0%) | 1 (1.3%) | 0 (0.0%) | 1 (0.3%) |  |  | |
| Blood pressure increased | 0 (0.0%) | 1 (1.3%) | 2 (2.6%) | 1 (1.3%) | 4 (1.3%) |  |  | |
| Body temperature increased | 4 (5.2%) | 5 (6.5%) | 1 (1.3%) | 2 (2.6%) | 12 (3.9%) |  |  | |
| C-reactive protein increased | 3 (3.9%) | 2 (2.6%) | 2 (2.6%) | 3 (3.8%) | 10 (3.2%) |  |  | |
| Gamma-glutamyltransferase increased | 3 (3.9%) | 0 (0.0%) | 1 (1.3%) | 1 (1.3%) | 5 (1.6%) |  |  | |
| Haemoglobin decreased | 1 (1.3%) | 0 (0.0%) | 0 (0.0%) | 0 (0.0%) | 1 (0.3%) |  |  | |
| Heart rate increased | 1 (1.3%) | 0 (0.0%) | 1 (1.3%) | 3 (3.8%) | 5 (1.6%) |  |  | |
| Hepatic enzyme increased | 0 (0.0%) | 0 (0.0%) | 0 (0.0%) | 1 (1.3%) | 1 (0.3%) |  |  | |
| Inflammatory marker increased | 0 (0.0%) | 0 (0.0%) | 1 (1.3%) | 0 (0.0%) | 1 (0.3%) |  |  | |
| Intraocular pressure increased | 0 (0.0%) | 1 (1.3%) | 0 (0.0%) | 0 (0.0%) | 1 (0.3%) |  |  | |
| Neutrophil count decreased | 1 (1.3%) | 2 (2.6%) | 1 (1.3%) | 1 (1.3%) | 5 (1.6%) |  |  | |
| Platelet count decreased | 1 (1.3%) | 0 (0.0%) | 0 (0.0%) | 0 (0.0%) | 1 (0.3%) |  |  | |
| Platelet count increased | 0 (0.0%) | 0 (0.0%) | 1 (1.3%) | 1 (1.3%) | 2 (0.6%) |  |  | |
| Pulse abnormal | 0 (0.0%) | 1 (1.3%) | 1 (1.3%) | 0 (0.0%) | 2 (0.6%) |  |  | |
| Sensory level abnormal | 1 (1.3%) | 0 (0.0%) | 1 (1.3%) | 0 (0.0%) | 2 (0.6%) |  |  | |
| Transaminases increased | 0 (0.0%) | 1 (1.3%) | 0 (0.0%) | 0 (0.0%) | 1 (0.3%) |  |  | |
| Weight decreased | 1 (1.3%) | 0 (0.0%) | 1 (1.3%) | 3 (3.8%) | 5 (1.6%) |  |  | |
| Weight increased | 3 (3.9%) | 3 (3.9%) | 2 (2.6%) | 1 (1.3%) | 9 (2.9%) |  |  | |
| White blood cell count decreased | 2 (2.6%) | 0 (0.0%) | 1 (1.3%) | 0 (0.0%) | 3 (1.0%) |  |  | |
|  | | | | | | | |  |
| **Metabolism and nutrition disorders** | | | | | | | |  |
|  | 17 (22.1%) | 22 (28.6%) | 17 (22.1%) | 17 (21.8%) | 73 (23.6%) |  |  | |
| Decreased appetite | 7 (9.1%) | 14 (18.2%) | 13 (16.9%) | 12 (15.4%) | 46 (14.9%) |  |  | |
| Dehydration | 0 (0.0%) | 0 (0.0%) | 0 (0.0%) | 2 (2.6%) | 2 (0.6%) |  |  | |
| Diabetes mellitus | 1 (1.3%) | 0 (0.0%) | 1 (1.3%) | 0 (0.0%) | 2 (0.6%) |  |  | |
| Electrolyte imbalance | 1 (1.3%) | 0 (0.0%) | 0 (0.0%) | 0 (0.0%) | 1 (0.3%) |  |  | |
| Fluid retention | 0 (0.0%) | 3 (3.9%) | 2 (2.6%) | 1 (1.3%) | 6 (1.9%) |  |  | |
| Hyperglycaemia | 2 (2.6%) | 0 (0.0%) | 0 (0.0%) | 1 (1.3%) | 3 (1.0%) |  |  | |
| Hyperkalaemia | 0 (0.0%) | 1 (1.3%) | 0 (0.0%) | 0 (0.0%) | 1 (0.3%) |  |  | |
| Hyperuricaemia | 0 (0.0%) | 0 (0.0%) | 0 (0.0%) | 1 (1.3%) | 1 (0.3%) |  |  | |
| Hypocalcaemia | 0 (0.0%) | 1 (1.3%) | 0 (0.0%) | 1 (1.3%) | 2 (0.6%) |  |  | |
| Hypokalaemia | 2 (2.6%) | 2 (2.6%) | 2 (2.6%) | 3 (3.8%) | 9 (2.9%) |  |  | |
| Hyponatraemia | 2 (2.6%) | 0 (0.0%) | 0 (0.0%) | 0 (0.0%) | 2 (0.6%) |  |  | |
| Increased appetite | 4 (5.2%) | 1 (1.3%) | 0 (0.0%) | 0 (0.0%) | 5 (1.6%) |  |  | |
| Iron deficiency | 0 (0.0%) | 0 (0.0%) | 0 (0.0%) | 1 (1.3%) | 1 (0.3%) |  |  | |
|  | | | | | | | |  |
| **Musculoskeletal and connective tissue disorders** | | | | | | | |  |
|  | 46 (59.7%) | 50 (64.9%) | 50 (64.9%) | 52 (66.7%) | 198 (64.1%) |  |  | |
| Arthralgia | 9 (11.7%) | 12 (15.6%) | 16 (20.8%) | 17 (21.8%) | 54 (17.5%) |  |  | |
| Back pain | 4 (5.2%) | 3 (3.9%) | 1 (1.3%) | 4 (5.1%) | 12 (3.9%) |  |  | |
| Bone callus excessive | 0 (0.0%) | 0 (0.0%) | 1 (1.3%) | 0 (0.0%) | 1 (0.3%) |  |  | |
| Bone pain | 24 (31.2%) | 19 (24.7%) | 25 (32.5%) | 30 (38.5%) | 98 (31.7%) |  |  | |
| Coccydynia | 0 (0.0%) | 1 (1.3%) | 0 (0.0%) | 0 (0.0%) | 1 (0.3%) |  |  | |
| Groin pain | 0 (0.0%) | 0 (0.0%) | 1 (1.3%) | 0 (0.0%) | 1 (0.3%) |  |  | |
| Growing pains | 0 (0.0%) | 0 (0.0%) | 0 (0.0%) | 1 (1.3%) | 1 (0.3%) |  |  | |
| Intervertebral disc protrusion | 0 (0.0%) | 1 (1.3%) | 0 (0.0%) | 0 (0.0%) | 1 (0.3%) |  |  | |
| Jaw disorder | 0 (0.0%) | 1 (1.3%) | 0 (0.0%) | 0 (0.0%) | 1 (0.3%) |  |  | |
| Joint swelling | 1 (1.3%) | 1 (1.3%) | 0 (0.0%) | 1 (1.3%) | 3 (1.0%) |  |  | |
| Mobility decreased | 0 (0.0%) | 0 (0.0%) | 1 (1.3%) | 0 (0.0%) | 1 (0.3%) |  |  | |
| Muscle spasms | 1 (1.3%) | 1 (1.3%) | 4 (5.2%) | 1 (1.3%) | 7 (2.3%) |  |  | |
| Muscle tightness | 0 (0.0%) | 1 (1.3%) | 0 (0.0%) | 0 (0.0%) | 1 (0.3%) |  |  | |
| Muscle twitching | 0 (0.0%) | 0 (0.0%) | 1 (1.3%) | 0 (0.0%) | 1 (0.3%) |  |  | |
| Muscular weakness | 2 (2.6%) | 3 (3.9%) | 1 (1.3%) | 2 (2.6%) | 8 (2.6%) |  |  | |
| Musculoskeletal chest pain | 0 (0.0%) | 1 (1.3%) | 0 (0.0%) | 0 (0.0%) | 1 (0.3%) |  |  | |
| Musculoskeletal disorder | 0 (0.0%) | 0 (0.0%) | 1 (1.3%) | 0 (0.0%) | 1 (0.3%) |  |  | |
| Musculoskeletal pain | 4 (5.2%) | 4 (5.2%) | 2 (2.6%) | 5 (6.4%) | 15 (4.9%) |  |  | |
| Myalgia | 17 (22.1%) | 21 (27.3%) | 18 (23.4%) | 17 (21.8%) | 73 (23.6%) |  |  | |
| Neck pain | 0 (0.0%) | 1 (1.3%) | 1 (1.3%) | 0 (0.0%) | 2 (0.6%) |  |  | |
| Osteitis | 0 (0.0%) | 1 (1.3%) | 0 (0.0%) | 0 (0.0%) | 1 (0.3%) |  |  | |
| Osteoarthritis | 1 (1.3%) | 0 (0.0%) | 0 (0.0%) | 0 (0.0%) | 1 (0.3%) |  |  | |
| Osteonecrosis | 0 (0.0%) | 1 (1.3%) | 0 (0.0%) | 0 (0.0%) | 1 (0.3%) |  |  | |
| Pain in extremity | 17 (22.1%) | 17 (22.1%) | 17 (22.1%) | 18 (23.1%) | 69 (22.3%) |  |  | |
| Pain in jaw | 1 (1.3%) | 0 (0.0%) | 0 (0.0%) | 0 (0.0%) | 1 (0.3%) |  |  | |
| Sensation of heaviness | 1 (1.3%) | 0 (0.0%) | 0 (0.0%) | 1 (1.3%) | 2 (0.6%) |  |  | |
| Systemic lupus erythematosus | 0 (0.0%) | 0 (0.0%) | 0 (0.0%) | 1 (1.3%) | 1 (0.3%) |  |  | |
|  | | | | | | | |  |
| **Neoplasms benign, malignant and unspecified (incl cysts and polyps)** | | | | | | | |  |
|  | 1 (1.3%) | 1 (1.3%) | 0 (0.0%) | 1 (1.3%) | 3 (1.0%) |  |  | |
| Metastases to meninges | 0 (0.0%) | 0 (0.0%) | 0 (0.0%) | 1 (1.3%) | 1 (0.3%) |  |  | |
| Tumour pain | 1 (1.3%) | 1 (1.3%) | 0 (0.0%) | 0 (0.0%) | 2 (0.6%) |  |  | |
|  | | | | | | | |  |
| **Nervous system disorders** | | | | | | | |  |
|  | 51 (66.2%) | 53 (68.8%) | 53 (68.8%) | 57 (73.1%) | 214 (69.3%) |  |  | |
| Ageusia | 2 (2.6%) | 1 (1.3%) | 0 (0.0%) | 4 (5.1%) | 7 (2.3%) |  |  | |
| Anosmia | 1 (1.3%) | 0 (0.0%) | 0 (0.0%) | 0 (0.0%) | 1 (0.3%) |  |  | |
| Aphonia | 0 (0.0%) | 0 (0.0%) | 0 (0.0%) | 1 (1.3%) | 1 (0.3%) |  |  | |
| Balance disorder | 0 (0.0%) | 1 (1.3%) | 1 (1.3%) | 1 (1.3%) | 3 (1.0%) |  |  | |
| Burning sensation | 2 (2.6%) | 0 (0.0%) | 0 (0.0%) | 0 (0.0%) | 2 (0.6%) |  |  | |
| Cerebral infarction | 1 (1.3%) | 0 (0.0%) | 0 (0.0%) | 0 (0.0%) | 1 (0.3%) |  |  | |
| Cerebrovascular spasm | 0 (0.0%) | 1 (1.3%) | 0 (0.0%) | 0 (0.0%) | 1 (0.3%) |  |  | |
| Cervicobrachial syndrome | 0 (0.0%) | 1 (1.3%) | 0 (0.0%) | 0 (0.0%) | 1 (0.3%) |  |  | |
| Convulsions local | 1 (1.3%) | 0 (0.0%) | 0 (0.0%) | 0 (0.0%) | 1 (0.3%) |  |  | |
| Coordination abnormal | 0 (0.0%) | 1 (1.3%) | 0 (0.0%) | 0 (0.0%) | 1 (0.3%) |  |  | |
| Disturbance in attention | 2 (2.6%) | 3 (3.9%) | 1 (1.3%) | 1 (1.3%) | 7 (2.3%) |  |  | |
| Dizziness | 1 (1.3%) | 2 (2.6%) | 0 (0.0%) | 1 (1.3%) | 4 (1.3%) |  |  | |
| Dysaesthesia | 0 (0.0%) | 0 (0.0%) | 3 (3.9%) | 1 (1.3%) | 4 (1.3%) |  |  | |
| Dysgeusia | 19 (24.7%) | 25 (32.5%) | 23 (29.9%) | 30 (38.5%) | 97 (31.4%) |  |  | |
| Formication | 0 (0.0%) | 1 (1.3%) | 0 (0.0%) | 1 (1.3%) | 2 (0.6%) |  |  | |
| Headache | 22 (28.6%) | 23 (29.9%) | 18 (23.4%) | 14 (17.9%) | 77 (24.9%) |  |  | |
| Hyperaesthesia | 2 (2.6%) | 2 (2.6%) | 2 (2.6%) | 0 (0.0%) | 6 (1.9%) |  |  | |
| Hypoaesthesia | 3 (3.9%) | 7 (9.1%) | 2 (2.6%) | 6 (7.7%) | 18 (5.8%) |  |  | |
| Hypogeusia | 0 (0.0%) | 2 (2.6%) | 1 (1.3%) | 1 (1.3%) | 4 (1.3%) |  |  | |
| Hypotonia | 0 (0.0%) | 2 (2.6%) | 2 (2.6%) | 0 (0.0%) | 4 (1.3%) |  |  | |
| Lethargy | 0 (0.0%) | 0 (0.0%) | 1 (1.3%) | 0 (0.0%) | 1 (0.3%) |  |  | |
| Loss of consciousness | 1 (1.3%) | 0 (0.0%) | 0 (0.0%) | 0 (0.0%) | 1 (0.3%) |  |  | |
| Memory impairment | 2 (2.6%) | 1 (1.3%) | 0 (0.0%) | 0 (0.0%) | 3 (1.0%) |  |  | |
| Migraine | 1 (1.3%) | 0 (0.0%) | 1 (1.3%) | 0 (0.0%) | 2 (0.6%) |  |  | |
| Neuralgia | 0 (0.0%) | 1 (1.3%) | 0 (0.0%) | 0 (0.0%) | 1 (0.3%) |  |  | |
| Neuropathy peripheral | 5 (6.5%) | 5 (6.5%) | 8 (10.4%) | 4 (5.1%) | 22 (7.1%) |  |  | |
| Neurotoxicity | 0 (0.0%) | 0 (0.0%) | 1 (1.3%) | 0 (0.0%) | 1 (0.3%) |  |  | |
| Orthostatic intolerance | 1 (1.3%) | 0 (0.0%) | 0 (0.0%) | 0 (0.0%) | 1 (0.3%) |  |  | |
| Paraesthesia | 10 (13.0%) | 9 (11.7%) | 9 (11.7%) | 15 (19.2%) | 43 (13.9%) |  |  | |
| Parosmia | 1 (1.3%) | 1 (1.3%) | 0 (0.0%) | 0 (0.0%) | 2 (0.6%) |  |  | |
| Peripheral motor neuropathy | 1 (1.3%) | 0 (0.0%) | 0 (0.0%) | 0 (0.0%) | 1 (0.3%) |  |  | |
| Peripheral sensory neuropathy | 5 (6.5%) | 3 (3.9%) | 3 (3.9%) | 2 (2.6%) | 13 (4.2%) |  |  | |
| Polyneuropathy | 19 (24.7%) | 17 (22.1%) | 20 (26.0%) | 14 (17.9%) | 70 (22.7%) |  |  | |
| Presyncope | 2 (2.6%) | 0 (0.0%) | 0 (0.0%) | 0 (0.0%) | 2 (0.6%) |  |  | |
| Restless legs syndrome | 1 (1.3%) | 0 (0.0%) | 2 (2.6%) | 0 (0.0%) | 3 (1.0%) |  |  | |
| Sciatica | 0 (0.0%) | 0 (0.0%) | 0 (0.0%) | 2 (2.6%) | 2 (0.6%) |  |  | |
| Sensory disturbance | 0 (0.0%) | 1 (1.3%) | 1 (1.3%) | 0 (0.0%) | 2 (0.6%) |  |  | |
| Sensory loss | 1 (1.3%) | 0 (0.0%) | 0 (0.0%) | 0 (0.0%) | 1 (0.3%) |  |  | |
| Speech disorder | 0 (0.0%) | 1 (1.3%) | 0 (0.0%) | 0 (0.0%) | 1 (0.3%) |  |  | |
| Tremor | 5 (6.5%) | 1 (1.3%) | 1 (1.3%) | 0 (0.0%) | 7 (2.3%) |  |  | |
|  | | | | | | | |  |
| **Psychiatric disorders** | | | | | | | |  |
|  | 17 (22.1%) | 25 (32.5%) | 23 (29.9%) | 27 (34.6%) | 92 (29.8%) |  |  | |
| Acute stress disorder | 1 (1.3%) | 0 (0.0%) | 0 (0.0%) | 0 (0.0%) | 1 (0.3%) |  |  | |
| Adjustment disorder | 0 (0.0%) | 0 (0.0%) | 1 (1.3%) | 0 (0.0%) | 1 (0.3%) |  |  | |
| Aggression | 0 (0.0%) | 0 (0.0%) | 0 (0.0%) | 1 (1.3%) | 1 (0.3%) |  |  | |
| Anxiety | 3 (3.9%) | 1 (1.3%) | 0 (0.0%) | 1 (1.3%) | 5 (1.6%) |  |  | |
| Binge eating | 0 (0.0%) | 0 (0.0%) | 1 (1.3%) | 0 (0.0%) | 1 (0.3%) |  |  | |
| Confusional state | 0 (0.0%) | 1 (1.3%) | 0 (0.0%) | 0 (0.0%) | 1 (0.3%) |  |  | |
| Depressed mood | 1 (1.3%) | 1 (1.3%) | 2 (2.6%) | 0 (0.0%) | 4 (1.3%) |  |  | |
| Depression | 0 (0.0%) | 0 (0.0%) | 2 (2.6%) | 2 (2.6%) | 4 (1.3%) |  |  | |
| Disorientation | 0 (0.0%) | 2 (2.6%) | 0 (0.0%) | 0 (0.0%) | 2 (0.6%) |  |  | |
| Emotional distress | 0 (0.0%) | 1 (1.3%) | 1 (1.3%) | 1 (1.3%) | 3 (1.0%) |  |  | |
| Initial insomnia | 0 (0.0%) | 1 (1.3%) | 0 (0.0%) | 0 (0.0%) | 1 (0.3%) |  |  | |
| Insomnia | 4 (5.2%) | 9 (11.7%) | 7 (9.1%) | 8 (10.3%) | 28 (9.1%) |  |  | |
| Mental disorder | 0 (0.0%) | 1 (1.3%) | 0 (0.0%) | 1 (1.3%) | 2 (0.6%) |  |  | |
| Mood altered | 2 (2.6%) | 0 (0.0%) | 1 (1.3%) | 2 (2.6%) | 5 (1.6%) |  |  | |
| Nervousness | 0 (0.0%) | 0 (0.0%) | 1 (1.3%) | 0 (0.0%) | 1 (0.3%) |  |  | |
| Panic attack | 1 (1.3%) | 0 (0.0%) | 0 (0.0%) | 0 (0.0%) | 1 (0.3%) |  |  | |
| Restlessness | 1 (1.3%) | 1 (1.3%) | 1 (1.3%) | 0 (0.0%) | 3 (1.0%) |  |  | |
| Sleep disorder | 8 (10.4%) | 11 (14.3%) | 10 (13.0%) | 12 (15.4%) | 41 (13.3%) |  |  | |
| Tearfulness | 1 (1.3%) | 0 (0.0%) | 0 (0.0%) | 0 (0.0%) | 1 (0.3%) |  |  | |
| Tension | 0 (0.0%) | 1 (1.3%) | 0 (0.0%) | 0 (0.0%) | 1 (0.3%) |  |  | |
|  | | | | | | | |  |
| **Renal and urinary disorders** | | | | | | | |  |
|  | 7 (9.1%) | 6 (7.8%) | 1 (1.3%) | 4 (5.1%) | 18 (5.8%) |  |  | |
| Chromaturia | 2 (2.6%) | 0 (0.0%) | 0 (0.0%) | 0 (0.0%) | 2 (0.6%) |  |  | |
| Dysuria | 2 (2.6%) | 3 (3.9%) | 1 (1.3%) | 0 (0.0%) | 6 (1.9%) |  |  | |
| Haematuria | 2 (2.6%) | 0 (0.0%) | 0 (0.0%) | 0 (0.0%) | 2 (0.6%) |  |  | |
| Nocturia | 1 (1.3%) | 2 (2.6%) | 0 (0.0%) | 1 (1.3%) | 4 (1.3%) |  |  | |
| Pollakiuria | 2 (2.6%) | 1 (1.3%) | 0 (0.0%) | 0 (0.0%) | 3 (1.0%) |  |  | |
| Polyuria | 0 (0.0%) | 0 (0.0%) | 0 (0.0%) | 1 (1.3%) | 1 (0.3%) |  |  | |
| Renal failure acute | 0 (0.0%) | 1 (1.3%) | 0 (0.0%) | 2 (2.6%) | 3 (1.0%) |  |  | |
| Renal pain | 1 (1.3%) | 0 (0.0%) | 0 (0.0%) | 0 (0.0%) | 1 (0.3%) |  |  | |
|  | | | | | | | |  |
| **Reproductive system and breast disorders** | | | | | | | |  |
|  | 8 (10.4%) | 15 (19.5%) | 11 (14.3%) | 10 (12.8%) | 44 (14.2%) |  |  | |
| Amenorrhoea | 0 (0.0%) | 1 (1.3%) | 0 (0.0%) | 1 (1.3%) | 2 (0.6%) |  |  | |
| Breast calcifications | 0 (0.0%) | 0 (0.0%) | 0 (0.0%) | 1 (1.3%) | 1 (0.3%) |  |  | |
| Breast discomfort | 1 (1.3%) | 0 (0.0%) | 0 (0.0%) | 0 (0.0%) | 1 (0.3%) |  |  | |
| Breast haematoma | 1 (1.3%) | 0 (0.0%) | 0 (0.0%) | 1 (1.3%) | 2 (0.6%) |  |  | |
| Breast induration | 0 (0.0%) | 0 (0.0%) | 1 (1.3%) | 0 (0.0%) | 1 (0.3%) |  |  | |
| Breast inflammation | 0 (0.0%) | 1 (1.3%) | 0 (0.0%) | 0 (0.0%) | 1 (0.3%) |  |  | |
| Breast pain | 3 (3.9%) | 5 (6.5%) | 4 (5.2%) | 3 (3.8%) | 15 (4.9%) |  |  | |
| Breast swelling | 0 (0.0%) | 1 (1.3%) | 0 (0.0%) | 0 (0.0%) | 1 (0.3%) |  |  | |
| Dysmenorrhoea | 1 (1.3%) | 1 (1.3%) | 0 (0.0%) | 0 (0.0%) | 2 (0.6%) |  |  | |
| Genital erosion | 1 (1.3%) | 0 (0.0%) | 0 (0.0%) | 0 (0.0%) | 1 (0.3%) |  |  | |
| Menopausal symptoms | 0 (0.0%) | 0 (0.0%) | 3 (3.9%) | 1 (1.3%) | 4 (1.3%) |  |  | |
| Menorrhagia | 0 (0.0%) | 2 (2.6%) | 0 (0.0%) | 0 (0.0%) | 2 (0.6%) |  |  | |
| Menstruation delayed | 0 (0.0%) | 1 (1.3%) | 0 (0.0%) | 0 (0.0%) | 1 (0.3%) |  |  | |
| Menstruation irregular | 0 (0.0%) | 1 (1.3%) | 4 (5.2%) | 0 (0.0%) | 5 (1.6%) |  |  | |
| Nipple disorder | 0 (0.0%) | 1 (1.3%) | 0 (0.0%) | 0 (0.0%) | 1 (0.3%) |  |  | |
| Pelvic pain | 0 (0.0%) | 1 (1.3%) | 0 (0.0%) | 1 (1.3%) | 2 (0.6%) |  |  | |
| Premenstrual syndrome | 0 (0.0%) | 1 (1.3%) | 0 (0.0%) | 0 (0.0%) | 1 (0.3%) |  |  | |
| Vaginal haemorrhage | 2 (2.6%) | 1 (1.3%) | 0 (0.0%) | 0 (0.0%) | 3 (1.0%) |  |  | |
| Vaginal inflammation | 0 (0.0%) | 0 (0.0%) | 0 (0.0%) | 1 (1.3%) | 1 (0.3%) |  |  | |
| Vulvovaginal burning sensation | 0 (0.0%) | 0 (0.0%) | 0 (0.0%) | 1 (1.3%) | 1 (0.3%) |  |  | |
| Vulvovaginal dryness | 0 (0.0%) | 0 (0.0%) | 1 (1.3%) | 1 (1.3%) | 2 (0.6%) |  |  | |
| Vulvovaginal pruritus | 1 (1.3%) | 1 (1.3%) | 0 (0.0%) | 1 (1.3%) | 3 (1.0%) |  |  | |
|  | | | | | | | |  |
| **Respiratory, thoracic and mediastinal disorders** | | | | | | | |  |
|  | 38 (49.4%) | 34 (44.2%) | 25 (32.5%) | 29 (37.2%) | 126 (40.8%) |  |  | |
| Acute respiratory distress syndrome | 0 (0.0%) | 1 (1.3%) | 0 (0.0%) | 0 (0.0%) | 1 (0.3%) |  |  | |
| Adenoidal disorder | 1 (1.3%) | 0 (0.0%) | 0 (0.0%) | 0 (0.0%) | 1 (0.3%) |  |  | |
| Allergic bronchitis | 1 (1.3%) | 0 (0.0%) | 0 (0.0%) | 0 (0.0%) | 1 (0.3%) |  |  | |
| Asthma | 0 (0.0%) | 0 (0.0%) | 0 (0.0%) | 1 (1.3%) | 1 (0.3%) |  |  | |
| Bronchospasm | 0 (0.0%) | 0 (0.0%) | 0 (0.0%) | 1 (1.3%) | 1 (0.3%) |  |  | |
| Chronic obstructive pulmonary disease | 0 (0.0%) | 0 (0.0%) | 0 (0.0%) | 1 (1.3%) | 1 (0.3%) |  |  | |
| Cough | 15 (19.5%) | 8 (10.4%) | 10 (13.0%) | 5 (6.4%) | 38 (12.3%) |  |  | |
| Dry throat | 1 (1.3%) | 0 (0.0%) | 0 (0.0%) | 0 (0.0%) | 1 (0.3%) |  |  | |
| Dysphonia | 3 (3.9%) | 2 (2.6%) | 0 (0.0%) | 0 (0.0%) | 5 (1.6%) |  |  | |
| Dyspnoea | 5 (6.5%) | 8 (10.4%) | 10 (13.0%) | 7 (9.0%) | 30 (9.7%) |  |  | |
| Dyspnoea exertional | 0 (0.0%) | 2 (2.6%) | 1 (1.3%) | 4 (5.1%) | 7 (2.3%) |  |  | |
| Epistaxis | 4 (5.2%) | 9 (11.7%) | 3 (3.9%) | 5 (6.4%) | 21 (6.8%) |  |  | |
| Hiccups | 0 (0.0%) | 0 (0.0%) | 1 (1.3%) | 0 (0.0%) | 1 (0.3%) |  |  | |
| Nasal discomfort | 0 (0.0%) | 0 (0.0%) | 1 (1.3%) | 0 (0.0%) | 1 (0.3%) |  |  | |
| Nasal dryness | 1 (1.3%) | 2 (2.6%) | 1 (1.3%) | 0 (0.0%) | 4 (1.3%) |  |  | |
| Nasal mucosal disorder | 1 (1.3%) | 1 (1.3%) | 0 (0.0%) | 0 (0.0%) | 2 (0.6%) |  |  | |
| Oropharyngeal blistering | 3 (3.9%) | 0 (0.0%) | 0 (0.0%) | 1 (1.3%) | 4 (1.3%) |  |  | |
| Oropharyngeal pain | 14 (18.2%) | 12 (15.6%) | 7 (9.1%) | 8 (10.3%) | 41 (13.3%) |  |  | |
| Painful respiration | 0 (0.0%) | 1 (1.3%) | 0 (0.0%) | 0 (0.0%) | 1 (0.3%) |  |  | |
| Pharyngeal erythema | 0 (0.0%) | 1 (1.3%) | 0 (0.0%) | 0 (0.0%) | 1 (0.3%) |  |  | |
| Pharyngeal oedema | 1 (1.3%) | 1 (1.3%) | 0 (0.0%) | 0 (0.0%) | 2 (0.6%) |  |  | |
| Pleural effusion | 0 (0.0%) | 1 (1.3%) | 0 (0.0%) | 0 (0.0%) | 1 (0.3%) |  |  | |
| Pneumothorax | 1 (1.3%) | 1 (1.3%) | 0 (0.0%) | 0 (0.0%) | 2 (0.6%) |  |  | |
| Productive cough | 2 (2.6%) | 1 (1.3%) | 0 (0.0%) | 0 (0.0%) | 3 (1.0%) |  |  | |
| Pulmonary embolism | 0 (0.0%) | 2 (2.6%) | 1 (1.3%) | 3 (3.8%) | 6 (1.9%) |  |  | |
| Rhinalgia | 1 (1.3%) | 0 (0.0%) | 0 (0.0%) | 0 (0.0%) | 1 (0.3%) |  |  | |
| Rhinorrhoea | 3 (3.9%) | 0 (0.0%) | 1 (1.3%) | 0 (0.0%) | 4 (1.3%) |  |  | |
| Throat irritation | 1 (1.3%) | 0 (0.0%) | 1 (1.3%) | 1 (1.3%) | 3 (1.0%) |  |  | |
| Throat tightness | 0 (0.0%) | 2 (2.6%) | 1 (1.3%) | 0 (0.0%) | 3 (1.0%) |  |  | |
|  | | | | | | | |  |
| **Skin and subcutaneous tissue disorders** | | | | | | | |  |
|  | 54 (70.1%) | 63 (81.8%) | 51 (66.2%) | 58 (74.4%) | 226 (73.1%) |  |  | |
| Acne | 1 (1.3%) | 4 (5.2%) | 0 (0.0%) | 1 (1.3%) | 6 (1.9%) |  |  | |
| Alopecia | 33 (42.9%) | 32 (41.6%) | 38 (49.4%) | 31 (39.7%) | 134 (43.4%) |  |  | |
| Angioedema | 0 (0.0%) | 1 (1.3%) | 0 (0.0%) | 0 (0.0%) | 1 (0.3%) |  |  | |
| Blister | 0 (0.0%) | 1 (1.3%) | 0 (0.0%) | 1 (1.3%) | 2 (0.6%) |  |  | |
| Blood blister | 0 (0.0%) | 1 (1.3%) | 0 (0.0%) | 0 (0.0%) | 1 (0.3%) |  |  | |
| Cold sweat | 0 (0.0%) | 0 (0.0%) | 1 (1.3%) | 0 (0.0%) | 1 (0.3%) |  |  | |
| Dermal cyst | 0 (0.0%) | 2 (2.6%) | 0 (0.0%) | 1 (1.3%) | 3 (1.0%) |  |  | |
| Dermatitis | 1 (1.3%) | 2 (2.6%) | 1 (1.3%) | 0 (0.0%) | 4 (1.3%) |  |  | |
| Dermatitis acneiform | 0 (0.0%) | 0 (0.0%) | 0 (0.0%) | 1 (1.3%) | 1 (0.3%) |  |  | |
| Dermatitis contact | 0 (0.0%) | 1 (1.3%) | 0 (0.0%) | 0 (0.0%) | 1 (0.3%) |  |  | |
| Dry skin | 7 (9.1%) | 14 (18.2%) | 9 (11.7%) | 8 (10.3%) | 38 (12.3%) |  |  | |
| Eczema | 0 (0.0%) | 3 (3.9%) | 0 (0.0%) | 2 (2.6%) | 5 (1.6%) |  |  | |
| Erythema | 5 (6.5%) | 11 (14.3%) | 6 (7.8%) | 5 (6.4%) | 27 (8.7%) |  |  | |
| Erythema multiforme | 0 (0.0%) | 1 (1.3%) | 0 (0.0%) | 0 (0.0%) | 1 (0.3%) |  |  | |
| Hyperhidrosis | 5 (6.5%) | 4 (5.2%) | 5 (6.5%) | 2 (2.6%) | 16 (5.2%) |  |  | |
| Intertrigo | 0 (0.0%) | 1 (1.3%) | 0 (0.0%) | 0 (0.0%) | 1 (0.3%) |  |  | |
| Nail bed inflammation | 0 (0.0%) | 0 (0.0%) | 0 (0.0%) | 1 (1.3%) | 1 (0.3%) |  |  | |
| Nail discolouration | 6 (7.8%) | 2 (2.6%) | 2 (2.6%) | 5 (6.4%) | 15 (4.9%) |  |  | |
| Nail disorder | 9 (11.7%) | 14 (18.2%) | 6 (7.8%) | 13 (16.7%) | 42 (13.6%) |  |  | |
| Nail dystrophy | 6 (7.8%) | 5 (6.5%) | 3 (3.9%) | 3 (3.8%) | 17 (5.5%) |  |  | |
| Nail toxicity | 0 (0.0%) | 2 (2.6%) | 1 (1.3%) | 0 (0.0%) | 3 (1.0%) |  |  | |
| Neurodermatitis | 0 (0.0%) | 1 (1.3%) | 0 (0.0%) | 0 (0.0%) | 1 (0.3%) |  |  | |
| Night sweats | 2 (2.6%) | 3 (3.9%) | 3 (3.9%) | 3 (3.8%) | 11 (3.6%) |  |  | |
| Onychalgia | 2 (2.6%) | 2 (2.6%) | 1 (1.3%) | 4 (5.1%) | 9 (2.9%) |  |  | |
| Onychoclasis | 3 (3.9%) | 10 (13.0%) | 4 (5.2%) | 7 (9.0%) | 24 (7.8%) |  |  | |
| Onycholysis | 1 (1.3%) | 1 (1.3%) | 2 (2.6%) | 3 (3.8%) | 7 (2.3%) |  |  | |
| Onychomadesis | 3 (3.9%) | 2 (2.6%) | 2 (2.6%) | 1 (1.3%) | 8 (2.6%) |  |  | |
| Pain of skin | 1 (1.3%) | 0 (0.0%) | 1 (1.3%) | 0 (0.0%) | 2 (0.6%) |  |  | |
| Palmar-plantar erythrodysaesthesia syndrome | 2 (2.6%) | 4 (5.2%) | 6 (7.8%) | 3 (3.8%) | 15 (4.9%) |  |  | |
| Petechiae | 1 (1.3%) | 0 (0.0%) | 0 (0.0%) | 0 (0.0%) | 1 (0.3%) |  |  | |
| Photosensitivity reaction | 0 (0.0%) | 0 (0.0%) | 0 (0.0%) | 1 (1.3%) | 1 (0.3%) |  |  | |
| Pigmentation disorder | 1 (1.3%) | 0 (0.0%) | 0 (0.0%) | 0 (0.0%) | 1 (0.3%) |  |  | |
| Pityriasis | 0 (0.0%) | 1 (1.3%) | 0 (0.0%) | 0 (0.0%) | 1 (0.3%) |  |  | |
| Pruritus | 3 (3.9%) | 8 (10.4%) | 3 (3.9%) | 5 (6.4%) | 19 (6.1%) |  |  | |
| Rash | 4 (5.2%) | 18 (23.4%) | 4 (5.2%) | 14 (17.9%) | 40 (12.9%) |  |  | |
| Rash macular | 0 (0.0%) | 2 (2.6%) | 0 (0.0%) | 0 (0.0%) | 2 (0.6%) |  |  | |
| Rash maculo-papular | 0 (0.0%) | 0 (0.0%) | 0 (0.0%) | 1 (1.3%) | 1 (0.3%) |  |  | |
| Rash pruritic | 0 (0.0%) | 1 (1.3%) | 0 (0.0%) | 0 (0.0%) | 1 (0.3%) |  |  | |
| Scar pain | 0 (0.0%) | 2 (2.6%) | 0 (0.0%) | 1 (1.3%) | 3 (1.0%) |  |  | |
| Skin burning sensation | 0 (0.0%) | 1 (1.3%) | 0 (0.0%) | 1 (1.3%) | 2 (0.6%) |  |  | |
| Skin disorder | 2 (2.6%) | 3 (3.9%) | 1 (1.3%) | 0 (0.0%) | 6 (1.9%) |  |  | |
| Skin exfoliation | 0 (0.0%) | 2 (2.6%) | 1 (1.3%) | 3 (3.8%) | 6 (1.9%) |  |  | |
| Skin fissures | 1 (1.3%) | 2 (2.6%) | 0 (0.0%) | 2 (2.6%) | 5 (1.6%) |  |  | |
| Skin fragility | 0 (0.0%) | 0 (0.0%) | 1 (1.3%) | 0 (0.0%) | 1 (0.3%) |  |  | |
| Skin hypertrophy | 1 (1.3%) | 1 (1.3%) | 0 (0.0%) | 0 (0.0%) | 2 (0.6%) |  |  | |
| Skin irritation | 0 (0.0%) | 0 (0.0%) | 1 (1.3%) | 1 (1.3%) | 2 (0.6%) |  |  | |
| Skin reaction | 0 (0.0%) | 1 (1.3%) | 0 (0.0%) | 0 (0.0%) | 1 (0.3%) |  |  | |
| Skin toxicity | 0 (0.0%) | 0 (0.0%) | 0 (0.0%) | 1 (1.3%) | 1 (0.3%) |  |  | |
| Skin ulcer | 0 (0.0%) | 0 (0.0%) | 0 (0.0%) | 1 (1.3%) | 1 (0.3%) |  |  | |
| Swelling face | 2 (2.6%) | 2 (2.6%) | 0 (0.0%) | 1 (1.3%) | 5 (1.6%) |  |  | |
| Urticaria | 0 (0.0%) | 0 (0.0%) | 1 (1.3%) | 2 (2.6%) | 3 (1.0%) |  |  | |
|  | | | | | | | |  |
| **Surgical and medical procedures** | | | | | | | |  |
|  | 0 (0.0%) | 0 (0.0%) | 0 (0.0%) | 1 (1.3%) | 1 (0.3%) |  |  | |
| Nail operation | 0 (0.0%) | 0 (0.0%) | 0 (0.0%) | 1 (1.3%) | 1 (0.3%) |  |  | |
|  | | | | | | | |  |
| **Vascular disorders** | | | | | | | |  |
|  | 32 (41.6%) | 32 (41.6%) | 21 (27.3%) | 28 (35.9%) | 113 (36.6%) |  |  | |
| Blood pressure fluctuation | 0 (0.0%) | 0 (0.0%) | 1 (1.3%) | 0 (0.0%) | 1 (0.3%) |  |  | |
| Circulatory collapse | 1 (1.3%) | 0 (0.0%) | 0 (0.0%) | 2 (2.6%) | 3 (1.0%) |  |  | |
| Deep vein thrombosis | 2 (2.6%) | 0 (0.0%) | 0 (0.0%) | 1 (1.3%) | 3 (1.0%) |  |  | |
| Flushing | 0 (0.0%) | 5 (6.5%) | 2 (2.6%) | 2 (2.6%) | 9 (2.9%) |  |  | |
| Haematoma | 1 (1.3%) | 2 (2.6%) | 0 (0.0%) | 0 (0.0%) | 3 (1.0%) |  |  | |
| Haemorrhage | 0 (0.0%) | 0 (0.0%) | 1 (1.3%) | 0 (0.0%) | 1 (0.3%) |  |  | |
| Hot flush | 14 (18.2%) | 18 (23.4%) | 13 (16.9%) | 15 (19.2%) | 60 (19.4%) |  |  | |
| Hypertension | 0 (0.0%) | 2 (2.6%) | 0 (0.0%) | 3 (3.8%) | 5 (1.6%) |  |  | |
| Hypotension | 4 (5.2%) | 2 (2.6%) | 1 (1.3%) | 1 (1.3%) | 8 (2.6%) |  |  | |
| Intra-abdominal haematoma | 1 (1.3%) | 0 (0.0%) | 0 (0.0%) | 0 (0.0%) | 1 (0.3%) |  |  | |
| Lymphoedema | 0 (0.0%) | 1 (1.3%) | 0 (0.0%) | 0 (0.0%) | 1 (0.3%) |  |  | |
| Peripheral coldness | 0 (0.0%) | 1 (1.3%) | 0 (0.0%) | 0 (0.0%) | 1 (0.3%) |  |  | |
| Phlebitis | 2 (2.6%) | 1 (1.3%) | 0 (0.0%) | 3 (3.8%) | 6 (1.9%) |  |  | |
| Subclavian vein thrombosis | 0 (0.0%) | 1 (1.3%) | 0 (0.0%) | 1 (1.3%) | 2 (0.6%) |  |  | |
| Thrombophlebitis | 11 (14.3%) | 5 (6.5%) | 4 (5.2%) | 3 (3.8%) | 23 (7.4%) |  |  | |
| Thrombophlebitis superficial | 0 (0.0%) | 1 (1.3%) | 0 (0.0%) | 0 (0.0%) | 1 (0.3%) |  |  | |
| Thrombosis | 1 (1.3%) | 1 (1.3%) | 1 (1.3%) | 2 (2.6%) | 5 (1.6%) |  |  | |
| Varicose vein | 1 (1.3%) | 0 (0.0%) | 1 (1.3%) | 0 (0.0%) | 2 (0.6%) |  |  | |
| Venous thrombosis | 0 (0.0%) | 1 (1.3%) | 0 (0.0%) | 1 (1.3%) | 2 (0.6%) |  |  | |
| Venous thrombosis limb | 1 (1.3%) | 0 (0.0%) | 1 (1.3%) | 0 (0.0%) | 2 (0.6%) |  |  | |
|  | | | | | | | |  |
